# Rewiring STAT signaling from the cell surface with Trikine immunotherapeutics

**DOI:** 10.1126/science.adx9954

**Published:** 2026-05-14

**Authors:** Grayson E. Rodriguez, Yang Zhao, Yoko Nishiga, Frank Peprah, Jiao Shen, Gita C. Abhiraman, Masato Ogishi, Chenyu Zhang, Justin Saco, Deepa Waghray, Mamatha Serasanambati, Leonel Torres, Brandon W. Simone, Leon Su, Steven C. Wilson, Aerin Yang, Qinli Sun, Lora Picton, Robert A. Saxton, Vidit Bhandarkar, Madeline J. Lee, Elizabeth Andrews, Hua Jiang, Matthias Obenaus, Michelle Yen, Tavus Atajanova, Catherine A. Blish, Stefani Spranger, E. John Wherry, Amanda Kirane, Antoni Ribas, David H. Raulet, Anusha Kalbasi, Stephanie K. Dougan, Michael Dougan, Julien Sage, K. Christopher Garcia

**Affiliations:** 1 Immunology Program, Stanford University, Stanford, CA, USA.; 2 Department of Molecular and Cellular Physiology, Stanford University School of Medicine, Stanford, CA, USA.; 3 Department of Radiation Oncology, Stanford University, Stanford, CA, USA; 4 Department of Pediatrics, Stanford University, Stanford, CA, USA; 5 Department of Genetics, Stanford University, Stanford, CA, USA; 6 Department of Medicine, Division of Gastroenterology, Massachusetts General Hospital, Boston, MA, USA.; 7 Department of Cancer Immunology and Virology, Dana-Farber Cancer Institute, Boston, MA, USA.; 8 Department of Immunology, Harvard Medical School, Boston, MA, USA.; 9 Division of Immunology and Molecular Medicine, Department of Molecular and Cell Biology, University of California, Berkeley, Berkeley, CA, USA.; 10 Division of Hematology-Oncology, Department of Medicine, University of California, Los Angeles, Los Angeles, CA, USA.; 11 Department of Molecular and Medical Pharmacology, University of California, Los Angeles, CA, USA.; 12 Department of Surgery, Division of Surgical Oncology, Stanford School of Medicine, Stanford University Medical Center, CA-USA-94305; 13 Institute for Immunology and Immune Health, University of Pennsylvania Perelman School of Medicine, Philadelphia, PA USA; 14 Center for Cellular Immunotherapies, University of Pennsylvania Perelman School of Medicine, Philadelphia, PA USA.; 15 Koch Institute at MIT, Cambridge, MA, USA.; 16 Department of Biology, MIT, Cambridge, MA, USA.; 17 Department of Medicine, Stanford University School of Medicine, Stanford, CA, USA; 18 Chan Zuckerberg Biohub, San Francisco, CA, USA; 19 Harvard Medical School, Boston, MA, USA.; 20 Ragon Institute of Mass General Hospital, MIT, and Harvard, Cambridge, MA, USA.; 21 Department of Systems Pharmacology and Translational Therapeutics, University of Pennsylvania Perelman School of Medicine, Philadelphia, PA, USA.; 22 Division of Hematology-Oncology, Department of Medicine, Jonsson Comprehensive Cancer Center, University of California Los Angeles, Los Angeles, CA, USA.; 23 Division of Surgical Oncology, Department of Surgery, University of California, Los Angeles, Los Angeles, CA, USA.; 24 Department of Radiation Oncology, Stanford University School of Medicine, Stanford, CA, USA.; 25 Stanford Center for Cancer Cell Therapy, Stanford University School of Medicine, Stanford, CA, USA.; 26 Stanford Cancer Institute, Stanford University School of Medicine, Stanford, CA, USA.; 27 Department of Structural Biology, Stanford University School of Medicine, Stanford, CA, USA.; 28 Howard Hughes Medical Institute, Stanford University School of Medicine, Stanford, CA, USA.

## Abstract

Cytokines dimerize two receptor chains to activate Janus kinases and STAT transcription factors that regulate immune cells but have therapeutic liabilities. We engineered “Trikines” to compel *cis* formation of three-chain cytokine receptor complexes at the cell surface that induce bespoke STAT transcriptional signaling programs optimized for therapeutic efficacy. Designed Trikines co-activated pSTAT5 and pSTAT3 signatures distinct from any natural cytokines, by assembling trimeric combinations of Interleukin-2 (IL-2), Interleukin-10 (IL-10), and Interleukin-21 (IL-21) receptors. An IL-2-based-Trikine restrained terminal differentiation of T cells, promoted stemness, and enhanced durability of tumor control without toxicity. Unexpectedly, an IL-10-based Trikine induced immune infiltration into poorly immunogenic tumors, showing striking efficacy in small cell lung cancer and pancreatic cancer models. Trikines obviate the need for cell engineering to customize STAT signatures for immunotherapy.

Cytokines modulate immune responses by dimerizing receptor pairs that activate Janus Kinases (JAKs), eliciting specific patterns of phosphorylated Signal Transducer and Activator of Transcription (pSTAT) proteins. These pSTAT profiles drive gene expression programs that determine cellular phenotypes and functions (1). For example, IL-2 is primarily a ‘pSTAT5’ cytokine, while IL-10 and IL-21 are ‘pSTAT3’ cytokines. While evolution has optimized these patterns for immune function, they are not necessarily ideal for therapeutic efficacy or tolerability (2).

Previously, we demonstrated that engineered or surrogate cytokines can attenuate or bias pSTAT activation, reducing pleiotropy (3-6). However, partial agonists only quantitatively modulate pSTAT activation without changing the qualitative profile—the specific STATs activated remain those naturally coupled to the cytokine receptor. Approaches that generate non-natural receptor heterodimers can elicit hybrid signals but often with attenuated strength, limiting functional outcomes (6, 7). Thus, a new strategy is needed to achieve robust, custom pSTAT signaling in natural immune cells.

Simultaneous strong pSTAT5/pSTAT3 activation has been shown to generate hybrid gene expression programs and new functionalities in engineered T cells (8, 9). We sought to engineer soluble agonists that would simultaneously induce pSTAT5 and pSTAT3, which are induced by the cytokines IL-2 (pSTAT5) and IL-21 and IL-10 (pSTAT3), respectively. IL-2 promotes T cell proliferation and survival and is FDA-approved to support adoptively transferred T cells (10), but persistent pSTAT5 signaling can drive terminal differentiation and exhaustion, limiting its utility (11-14). IL-21 and IL-10 signal via pSTAT3 and promote stem-like, less exhausted T cells with minimal proliferation (15-20). Despite promising biology, their natural signaling profiles are not optimized for maximal therapeutic benefit.

Here, we present a cytokine engineering strategy to rewire pSTAT signaling via three-chain signaling entities, or “Trikines.” Each Trikine is comprised of a “base” cytokine linked to binder to recruit a third receptor chain, enabling new STAT activation combinations. Trikines restrict dual STAT activation to single cells, overcoming the pleiotropy and toxicity of cytokine combinations. For example, an IL-2–based Trikine recruits the pSTAT3-associated IL-21Rα to the IL-2Rβ/γ_c_ complex, generating robust pSTAT3 alongside pSTAT5. Conversely, an IL-10–based Trikine recruits IL-2Rβ, conferring a pSTAT5 proinflammatory signature. Trikines thus offer a general strategy to confer custom signaling outputs to natural cytokines without genetic modification of T cells.

## Trikines exhibit AND-gated pSTAT induction

We deployed a “mix-and-match” engineering strategy using IL-2, IL-21, IL-10, and their respective receptors to create Trikines with differential levels of pSTAT5 and pSTAT3 co-induction followed by testing their functional properties. This represents an alternative strategy to simply fusing the cytokines together into a tandem molecule, where only a subset of cells expressing all receptors of the paired cytokines would receive the dual pSTAT5/pSTAT3 signal (21). Instead, Trikines actions are restricted to cells co-expressing three receptors to simultaneously deliver both pSTAT5 and pSTAT3 from one ternary signaling complex on a cell autonomous basis. With these design goals in mind, we created three Trikines, each with a “base cytokine” (IL-2, IL-21, IL-10) fused to a non-signaling binder to recruit an additional receptor to the signaling complex. Each of these cytokines has been explored as a cancer immunotherapeutic, but each is associated with distinct limitations and liabilities.

**Fig. 1.**
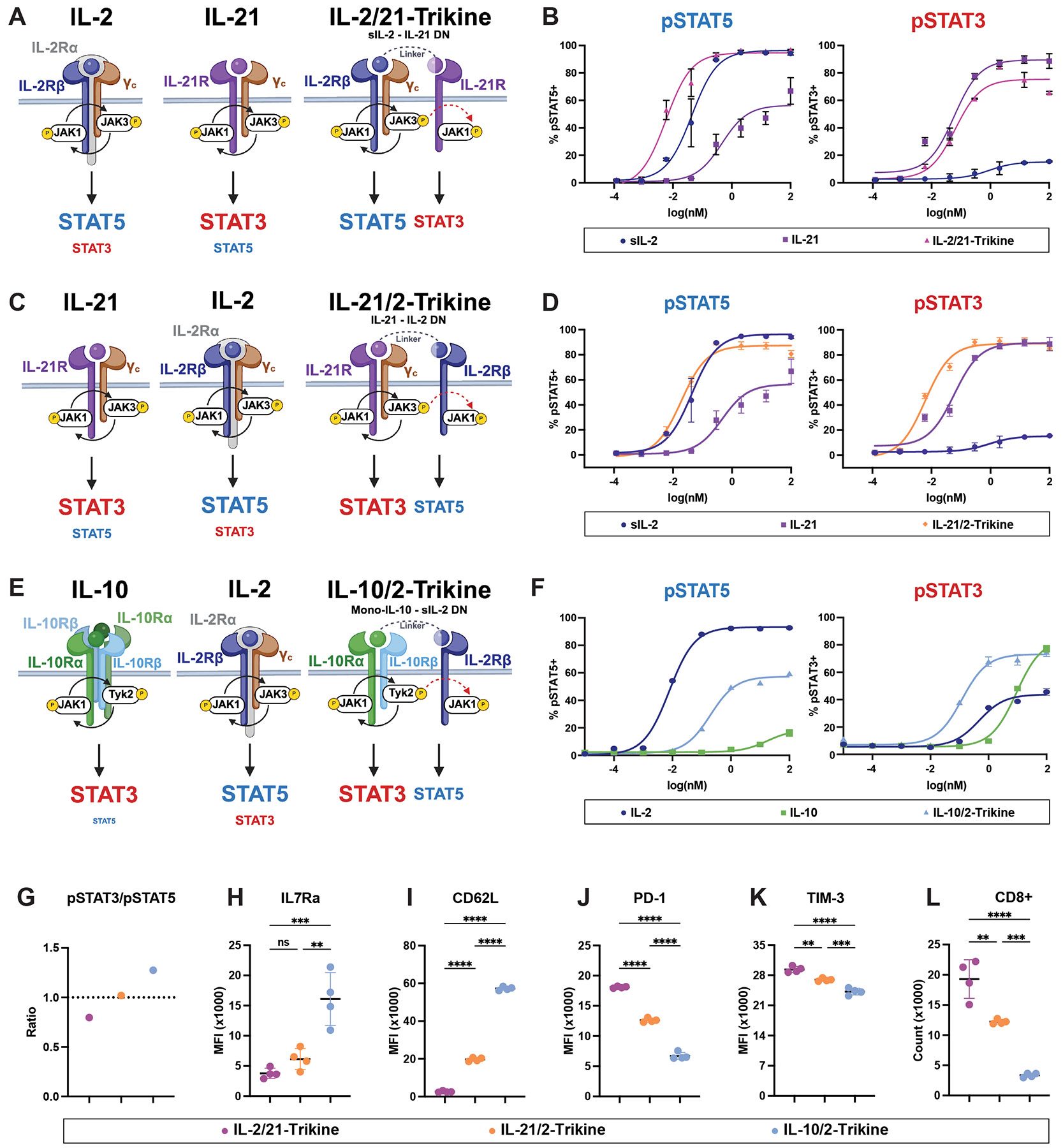
Trikines rebalance STAT3/STAT5 with differential effects on T cell stemness. (**A-B**) IL-2, IL-21, and IL-2/21-Trikine pSTAT signaling schematic (A) and dose-response on mouse CD8+ T cells (B). (**C-D**) IL-21, IL-2, and IL-21/2-Trikine pSTAT signaling schematic (C) and dose-response on mouse CD8+ T cells (D). (**E-F**) IL-10, IL-2, and IL-10/2-Trikine pSTAT signaling schematic (E) and dose-response on mouse CD8+ T cells (F). Values in (B, D, F) represent the percentage of CD8+ T cells that are pSTAT5+ (left) or pSTAT3+ (right). Errors bars indicate mean ± standard deviation (SD) of duplicate or triplicate wells. (**G**) The ratios of pSTAT3+ to pSTAT5+ CD8+ T cells following stimulation by IL-2/21-, IL-21/2-, or IL-10/2-Trikine for 15 minutes at Emax. (**H-L**) Pmel CD8+ T cells were activated with gp100 for 5 days and then starved overnight. Cells were then restimulated with 10 ng/mL anti-CD3 antibody and 10 nM indicated Trikines for 48 hours. MFI of IL7Ra (H), CD62L (I), PD-1 (J) and TIM-3 (K). CD8+ cell count (L). Results display 4 technical replicates and are representative of 2 independent experiments (B, D, F, G-L).

In one iteration, we created an IL-2/21-Trikine to endow the STAT5 cytokine IL-2 with enhanced STAT3 signaling. IL-2 signals principally through pSTAT5, activating pSTAT3 to a lesser degree. IL-21, another common gamma chain (γ_c_) cytokine family member, signals principally through pSTAT3, with a minor pSTAT5 signature We used a previously reported ‘super IL-2’ (sIL-2), engineered for high affinity to IL-2Rβ (22), to a mouse IL-21Rα binder (Fig. 1A). The IL-21Rα binder is a mouse IL-21 mutant that retains high affinity binding to IL-21Rα but is devoid of binding to γ_c,_ and thus incapable of signaling. We identified that Q121L fully eliminates the pSTAT3 signaling of IL-21 relative to wildtype (WT) IL-21 (Fig. S1A-C) (23). We thus used Q121L (referred to as IL-21 DN for dominant-negative) as the IL-21Rα binding module of our mouse-reactive IL-2/21-Trikine (Fig. 1A). This design has attributes of unidirectional AND gated IL-21 signaling (i.e., IL-21Rα is only activated in the presence of IL-2Rβ/γ_c_, whereas IL-2 signaling is not dependent on the presence of IL-21Rα). The IL-2/21-Trikine displayed equivalent pSTAT5 signaling compared to sIL-2 and ~84% E_max_ pSTAT3 signaling compared to IL-21, confirming rebalancing of pSTAT3 and pSTAT5 signaling relative to sIL-2 and murine IL-21 treatment of murine T cells (Fig. 1B).

In a second iteration, we created an IL-21/2-Trikine, in which we linked mouse IL-21 as the base cytokine to a non-agonist, DN form of sIL-2, that has high affinity for IL-2Rβ, which contains a previously reported set of mutations, RETR (L18R, Q22E, Q126T, and S130R) that abolish binding to γ_c_ (Fig. 1C) (24). The IL-21/2-Trikine induced equivalent pSTAT3 Emax as IL-21 and ~91% pSTAT5 Emax of sIL-2 in *in vitro* signaling assays (Fig. 1D).

In a third iteration, created an IL-10/2-Trikine, in which we used a previously reported monomeric form of human IL-10 (mono-IL-10), which primarily induces a pSTAT3 signal on both human and mouse cells, as the base cytokine (Fig. 1E). Mono-IL-10 signals via dimerizing of IL-10Rα and IL-10Rβ, and it has mutations that enhance its affinity to IL-10Rβ compared to WT (4). To add pSTAT5 induction to the pSTAT3 signaling of mono-IL-10, we used the same sIL-2 DN as in the IL-21/2-Trikine to recruit IL-2Rβ to the mono-IL-10/IL-10Rα/β signaling complex (Fig. 1E). Like the IL-21/2-Trikine, the IL-10/2-Trikine elicited robust pSTAT3 but more moderate pSTAT5 (~62% Emax of sIL-2) in murine T cells in a dose-dependent manner (Fig. 1F).

The ratios of the percentage of CD8+ T cells staining positive for pSTAT3 to those positive for pSTAT5 revealed differential balances of pSTAT3/pSTAT5 ratios among the Trikines (Fig. 1G). *In vitro* culture of CD8+ T cells demonstrated that pSTAT3/pSTAT5 levels are positively associated with expression of stemness markers (Fig. 1H-I) and negatively associated with proliferation and exhaustion marker expression (Fig. 1J-L). These phenotypic differences *in vitro* motivated us to further investigate the IL-2/21- and IL-10/2- Trikines, which have reciprocal pSTAT3/pSTAT5 balances (Fig. 1G).

## IL-2/21-Trikine potentiates pSTAT3 promoted genes

**Fig. 2.**
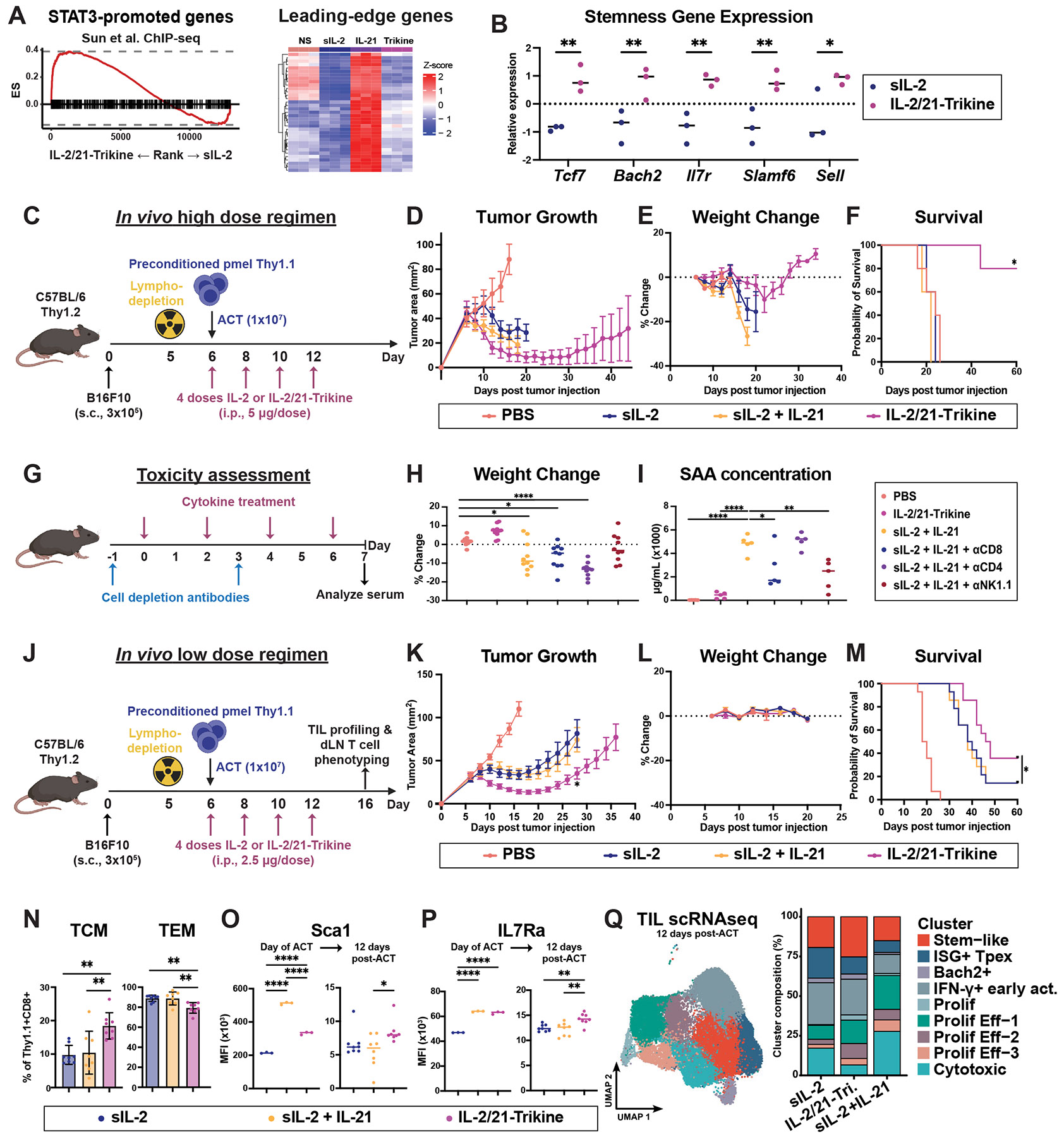
IL-2/21-Trikine has an enhanced pSTAT3 transcriptional profile and improves upon the safety and efficacy of cytokine therapy. (**A-B**) Mouse CD8+ T cells were treated with indicated cytokines for 24 hours prior to RNA isolation for bulk RNAseq analysis; (A) Geneset enrichment analysis (GSEA) for DEGs between IL-2/21-Trikine-treated vs sIL2-treated mouse CD8+ T cells (left) and heatmap of leading-edge genes (right). The DEG ranks were projected onto two gene sets defined based on a previously published ChIP-seq data (18). pSTAT3-promoted genes, genes with stronger ChIP peaks in STAT3-WT mice. (B) Relative expression of stemness genes *Tcf7*, *Bach2*, *Il7r*, *Slamf6*, and *Sell* between mouse CD8+ T cells treated with either sIL-2 or IL-2/21-Trikine. Results from one independent experiment with 3 technical replicates. (**C-F**), C57BL/6 mice bearing established s.c. B16F10 tumors received intravenous (i.v.) adoptive cell transfer (ACT) of pmel CD8+ T cells (1 x 10^7^) that were preconditioned for 7 days with sIL-2, sIL-2 + IL-21, or IL-2/21-Trikine on day 5, followed by i.p. injections of cytokine (5 μg functional cytokine) every other day until day 12 (n = 5 animals); the experimental timeline (C), average tumor growth curves (D), average weight change curves (E), and survival curves (F). Results from one independent experiment. (**G-I**) Healthy C57BL/6 mice were dosed with 10 μg functional dose of sIL-2, IL-21, sIL-2 + IL-21, IL-2/21-Trikine or PBS every other day for four total doses (n = 10 mice); experimental timeline (G) percent weight change at study endpoint (H), serum amyloid A concentrations at study endpoint (I). Results are combined from 2 independent experiments. (**J-M**), C57BL/6 mice bearing established s.c. B16F10 tumors received intravenous (i.v.) adoptive cell transfer (ACT) of pmel CD8+ T cells (1 x 10^7^) that were preconditioned for 7 days with sIL-2, sIL-2 + IL-21, or IL-2/21-Trikine on day 5, followed by i.p. injections of cytokine (2.5 μg functional cytokine) every other day until day 12 (n = 14/15 animals); the experimental timeline (J), average tumor growth curves (K), average weight change curves (L), and survival curves (M). Results are combined from 2 independent experiments. (**N**) Proportions of pmel T cell memory populations, TCM and TEM in the draining lymph node 16 days post tumor injection from mice receiving low dose regimen (J-M) (n = 8). Results representative of 2 independent experiments. (**O-P**) MFI of Sca1 (O) and IL7Ra (P) cells in the draining lymph node 12 days post ACT compared to on the day of ACT from mice receiving low dose regimen (J) (n = 8). Results representative of 2 independent experiments. (**Q**) Intratumoral, live Thy1.1+CD8+ cells were sorted from dissociated tumor 12 days after ACT as in (J) and sent for scRNA-seq. 8 transcriptionally distinct clusters are seen by UMAP (left) and cluster composition by treatment is displayed (right). scRNAseq data is from one independent experiment. Schematics in C, G, and J created using BioRender.com.

We performed bulk RNA-seq to assess the pSTAT3-driven signature induced by the IL-2/21-Trikine, confirming its distinct transcriptional profile from sIL-2 or IL-21 alone or in combination (Fig. S2A-E). The IL-2/21-Trikine better supports T-cell cycling gene pathways, while more weakly inducing p53-pathway genes, which is associated with T cell exhaustion (25, 26). Analyzing the differentially expressed genes (DEGs) within STAT family TFs via gene set enrichment analysis (GSEA) confirmed enrichment of STAT3 target gene transcription by the IL-2/21-Trikine (Fig. S2F), and gene ontology showed a significant enrichment of pSTAT3-promoted genes and suppression of STAT3-inhibited genes (Fig. 2A, Fig. S2G) (18). Moreover, stemness marker gene expression was significantly enhanced by the IL-2/21-Trikine relative to sIL-2 (Fig. 2B). These data demonstrate that the IL-2/21-Trikine promotes a distinct transcriptional signature from IL-2 alone and sIL-2 + IL-21 together, and, importantly, induces a pSTAT3 gene signature including downstream stemness-associated genes.

## *In vivo* efficacy and toxicity benchmarking the IL-2/21-Trikine

We directly compared the *in vivo* efficacy and toxicity of the IL-2/21-Trikine with sIL-2 alone or sIL-2 + IL-21, which remain clinically limited by dose-limiting toxicities. Although combined IL-2 and IL-21 signaling has been pursued for cancer immunotherapy, its clinical deployment has been precluded by toxicity of the individual cytokines (27). High-dose IL-2 produces durable responses in metastatic renal cell carcinoma and melanoma (28), but causes severe vascular leak syndrome and cytokine storm (29). Engineered IL-2 variants, including β/γ-biased molecules such as Super-2, remain constrained by toxicity (30-32), while IL-21 development was halted following dose-limiting toxicity in a Phase II melanoma trial (33).

To assess therapeutic index, we performed dose-finding studies using a well-established adoptive cell transfer (ACT) model. Thy1.1^+^ pmel T cells were preconditioned with gp100 peptide and cytokine and transferred into irradiated B16F10 tumor–bearing mice (Fig. 2C, S3A). Mice then received four intraperitoneal doses (5 μg) of mouse serum albumin–fused cytokine. To limit toxicity, mice receiving sIL-2– or sIL-2 + IL-21–preconditioned cells were treated with sIL-2 alone, whereas mice receiving IL-2/21-Trikine–preconditioned cells received Trikine dosing. Although all treatments delayed tumor growth relative to PBS controls (Fig. 2D, S3B), only IL-2/21-Trikine was well tolerated (Fig. 2E). sIL-2 and sIL-2 + IL-21 caused profound weight loss and conferred no survival benefit over PBS (Fig. 2F). In contrast, IL-2/21-Trikine achieved 80% long-term survival.

The maximum tolerated dose of sIL-2 was 10 μg, as higher doses (20–50 μg) significantly reduced survival (Fig. S3C-G). By contrast, IL-2/21-Trikine was tolerated up to 50 μg, representing a fivefold increase in therapeutic index. In healthy male and female mice, sIL-2 and sIL-2 + IL-21 induced splenomegaly and weight loss, while the combination markedly increased serum IFN-γ and impaired locomotion (Fig. S4A-I). Histopathology revealed substantial inflammation in liver, lung, and pancreas exclusively in the sIL-2 + IL-21 group (Fig. S4J-K). These data establish that IL-2/21-Trikine markedly reduces cytokine-associated systemic toxicity.

## IL-2/21-Trikine exerts a protective effect on toxicity through limited effects on NK cells *in vivo*

To define the cellular drivers of sIL-2 + IL-21 toxicity, we combined cytokine treatment with cell-type–specific depletion (Fig. 2G). NK cell depletion rescued weight loss and significantly reduced serum amyloid A (Fig. 2H-I, S5L), identifying NK cells as a principal mediator of toxicity (34, 35). The effects of sIL-2 and IL-21 on NK cell proliferation and cytotoxicity have been well documented (36-38). We therefore directly compared NK cell responses following IL-2/21-Trikine versus sIL-2 + IL-21 treatment *in vivo*. Mice received three cytokine doses, including two Trikine concentrations (Fig. S4M), and splenic NK cells were analyzed.

IL-2/21-Trikine did not increase NK cell abundance, proliferation (Ki67), or maturation (CD11b/CD27) (Fig. S4N-P). In contrast, sIL-2 and sIL-2 + IL-21 robustly upregulated NK activation markers Sca-1 and CD69 and enhanced IFN-γ production upon *ex vivo* restimulation (Fig. S4Q-S). Notably, IL-2/21-Trikine reduced IFN-γ secretion below baseline. Although IL-2/21-Trikine modestly increased NK cytotoxicity relative to untreated controls, this activity remained significantly lower than that induced by sIL-2 or sIL-2 + IL-21 (Fig. S4T).

These results establish NK cells as dominant drivers of sIL-2–associated toxicity, consistent with prior reports (39), and of toxicity induced by sIL-2 + IL-21. In the pmel ACT model, T cells likely contribute cooperatively, as sIL-2 and sIL-2 + IL-21 caused rapid deterioration despite dosing at only half the maximum tolerated dose of sIL-2 (Fig. 2C-F, S3C-F). Collectively, these data demonstrate that IL-2/21-Trikine enables potent *in vivo* efficacy while actively protecting against cytokine-driven toxicity, even at substantially elevated doses.

## IL-2/21-Trikine slows tumor growth and maintains T cell stemness *in vivo*

To directly compare IL-2/21-Trikine with sIL-2 and sIL-2 + IL-21, we applied a low-dose ACT regimen (Fig. 2J). This regimen was well tolerated across all groups, but only IL-2/21-Trikine significantly suppressed tumor growth relative to sIL-2 (Fig. 2K-L, S5A) and conferred a durable survival advantage compared to both sIL-2 and sIL-2 + IL-21 (Fig. 2M).

Draining lymph nodes (dLN) and tumors were harvested 12 days after ACT (Fig. S5B). Antigen-specific Thy1.1+CD8+ T cells from sIL-2– and sIL-2 + IL-21–treated mice were skewed toward a differentiated effector memory (TEM; CD62L–CD44+) phenotype, whereas IL-2/21-Trikine treatment preserved a significantly larger central memory (TCM; CD62L+CD44+) population in the dLN (Fig. 2N, S5C). Consistent with this phenotype, only IL-2/21-Trikine–treated pmel cells retained elevated expression of stemness-associated markers after tumor infiltration relative to their pre-transfer state (Fig. 2O-P). These data demonstrate that IL-2/21-Trikine restrains differentiation and preserves T cell stemness *in vivo*.

We next asked whether the distinct stemness program induced by IL-2/21-Trikine persisted within intratumoral T cells. Single-cell RNA-seq of live Thy1.1+CD8+ tumor-infiltrating lymphocytes isolated 12 days after ACT identified eight transcriptionally distinct clusters (Fig. 2Q, S6D-E). These included a Stem-like cluster (*Tcf7*, *Il7r*, *Ccr7*, *Bcl2*), a Bach2+ early-differentiation cluster (*Bach2*, *Foxo1*), an interferon-stimulated progenitor-exhausted (ISG+ Tpex) cluster, and multiple activated and cytotoxic effector subsets (Fig. S5E).

Cluster composition differed markedly by treatment. IL-2/21-Trikine–treated tumors were enriched for the Stem-like and *Bach2*+ clusters, consistent with maintenance of a minimally differentiated transcriptional state. In contrast, sIL-2 treatment reduced these populations and increased representation of the ISG+ Tpex cluster, indicating an alternative interferon-driven differentiation trajectory. Terminally differentiated cytotoxic T cells expressing *Pdcd1*, *Havcr2*, and *Entpd1* were least frequent in IL-2/21-Trikine–treated tumors. These intratumoral differentiation patterns mirrored those observed in the dLN, reinforcing that IL-2/21-Trikine sustains a less differentiated T cell pool *in vivo* compared to sIL-2 or sIL-2 + IL-21.

## Human-reactive IL-2/21-Trikine enhances T cell persistence and cytotoxicity *in vitro*

**Fig. 3.**
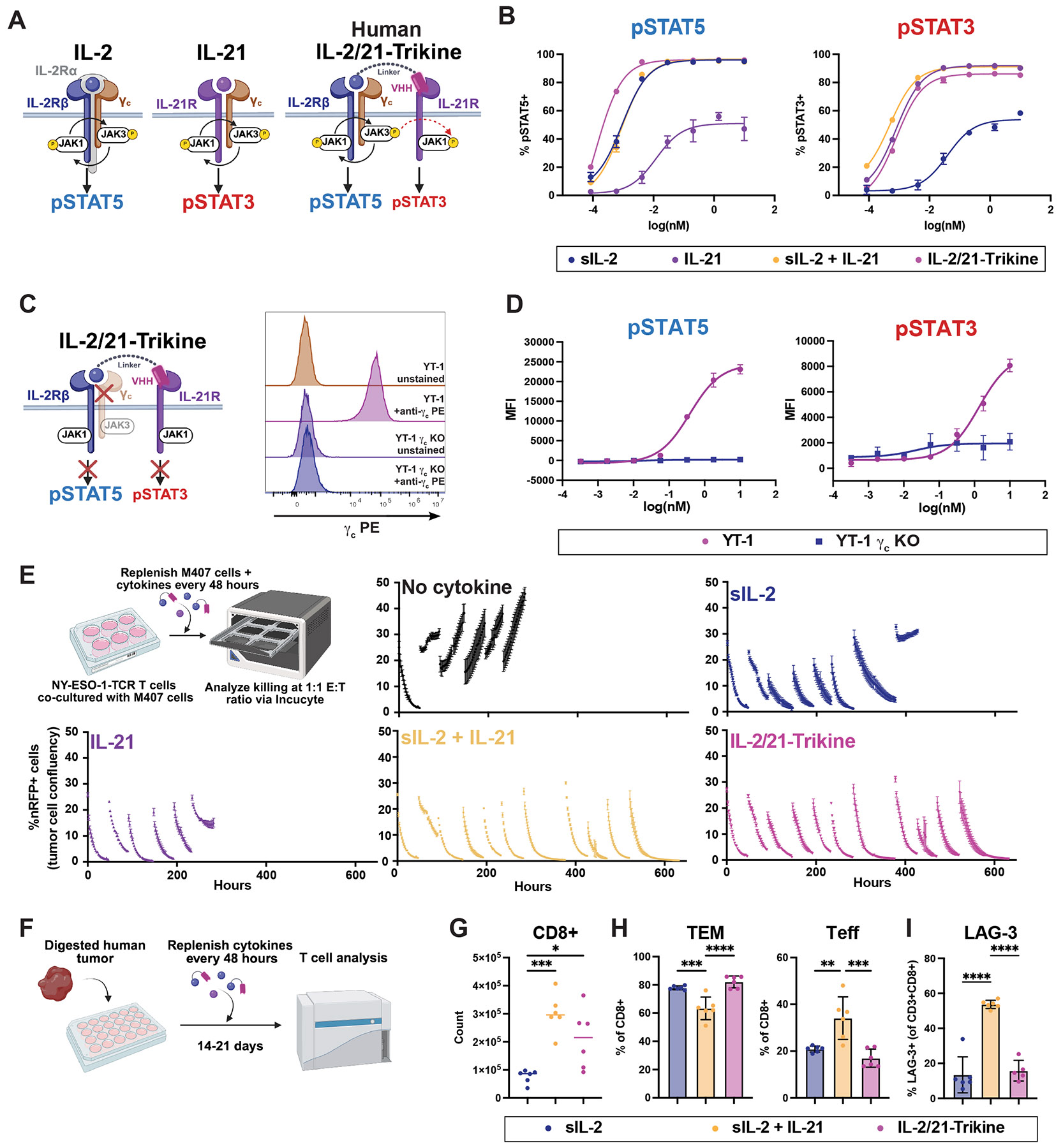
Human-reactive IL-2/21-Trikine enhances T cell persistence and cytotoxicity *in vitro*. (**A**) Schematic of IL-2, IL-21, and human-reactive IL-2/21-Trikine pSTAT signaling. (**B**) Dose-dependent pSTAT signaling of IL-2, IL-21, and human-reactive IL-2/21-Trikine on human CD8+ T cells. Errors bars indicate mean ± standard deviation (SD) of duplicate or triplicate wells. MFI, mean fluorescence intensity. Results are representative of 2 independent experiments. (**C**) Diagram of testing for human IL-2/21-Trikine signal on γ_c_ knock-out YT-1 cells (left) and flow cytometry staining for γ_c_ WT and knock-out YT-1 cells (right). (**D**) Human-reactive IL-2/21-Trikine pSTAT signaling in WT and γ_c_ knock-out YT-1 cells. Errors bars indicate mean ± standard deviation (SD) of duplicate or triplicate wells. Results are representative of 2 independent experiments. (**E**) Schematic of *in vitro* repeated antigen challenge (RAC) experiment (top left) and target cell killing as measured by IncuCyte after 11 rounds of RAC (right). Results are representative of 2 independent experiments. Error bars represent mean ± SD. (**F**) Schematic of human melanoma TIL experiment. (**G-I**) CD8+ T cell counts (G) , percentage of CD8+ T cells with TEM or Teff phenotype (H), and percentage of CD3+CD8+ cells that are LAG-3+ (I) from human melanoma TILs treated with sIL-2, sIL-2 + IL-21, or IL-2/21-Trikine for 14 (G-H) or 21 (I) days. Errors bars indicate mean ± standard deviation (SD) of 4 replicate wells. Results are representative of 2 independent experiments. Schematics in A, C, E, and F created using BioRender.com.

Given the robust efficacy and reduced toxicity of the mouse-reactive IL-2/21-Trikine, we engineered a human-reactive variant. Because sIL-2 is cross-reactive, only a human IL-21Rα–binding domain was required (Fig. 3A). An anti–IL-21Rα VHH was isolated from a synthetic yeast display library (Fig. S6A) (40), confirmed to bind IL-21Rα–expressing YT-1 cells (Fig. S6B), and shown by SPR to bind human IL-21Rα with 17.07 nM affinity (Fig. S6C). Fusion of this VHH to sIL-2 via a 3–amino acid (GSG) linker yielded optimal signaling in human CD8+ T cells, with equivalent pSTAT5 Emax and reduced EC50 compared to sIL-2, and ~93% of IL-21–induced pSTAT3 Emax (Fig. 3B). Thus, the human-reactive IL-2/21-Trikine faithfully recapitulates the signaling profile of the mouse-reactive molecule.

To define the signaling mechanism underlying enhanced pSTAT3, we tested whether IL-21Rα was activated via JAK1 or JAK3. After validating Trikine-induced pSTAT5 and pSTAT3 in YT-1 cells (Fig. S6D), we assessed signaling in γc knockout YT-1 cells (Fig. 3C). Loss of γc completely abrogated IL-2/21-Trikine signaling, identifying γc as a central signaling hub for coordinated activation of IL-2Rβ and IL-21Rα (Fig. 3D).

We next evaluated the capacity of the human-reactive IL-2/21-Trikine to sustain T cell function under chronic stimulation. In repeated antigen challenge assays (Fig. 3E), IL-2/21-Trikine best preserved T cell proliferation and cytotoxicity across multiple re-challenges, outperforming sIL-2. In a second chronic stimulation model (Fig. S6E), IL-2/21-Trikine significantly enriched IFN-γ+ and TNFα+ single-positive T cells over three weeks, and uniquely expanded IFN-γ+TNFα+ double-positive cells (Fig. S6F-G).

Finally, we assessed the translational potential of IL-2/21-Trikine for expansion of melanoma patient–derived tumor-infiltrating lymphocytes (TILs), benchmarked against the FDA-approved IL-2–based protocol (41) (Fig. 3F). Both sIL-2 + IL-21 and IL-2/21-Trikine enhanced CD8+ T cell expansion relative to sIL-2 alone (Fig. 3G). However, sIL-2 + IL-21 skewed cultures toward terminal differentiation, whereas sIL-2 and IL-2/21-Trikine preserved larger TEM and smaller effector populations (Fig. 3H). Moreover, sIL-2 + IL-21 significantly increased expression of the exhaustion marker LAG-3, while IL-2/21-Trikine did not (Fig. 3I) (42). Collectively, these data establish IL-2/21-Trikine as a potent, less toxic alternative for sustaining human T cell persistence and function, with clear advantages for ACT and TIL-based therapies.

## IL-10/2-Trikine reprograms TILs for enhanced antitumor efficacy in melanoma

IL-10 has been evaluated clinically across multiple solid tumor indications but has shown limited efficacy (20, 43). We therefore asked whether introduction of a STAT5 signal could potentiate its antitumor activity while avoiding additional toxicity, prompting evaluation of the IL-10/2-Trikine *in vitro* and *in vivo*. *In vitro*, the IL-10/2-Trikine significantly increased the frequency of SCA-1^+^CD62L^+^ T cells relative to all comparator conditions (Fig. S7A-B), consistent with enrichment of a stem-like T cell phenotype. Although the IL-10/2-Trikine did not enhance T cell proliferation (Fig. S7C), it upregulated expression of the IL-2–responsive marker CD44 (Fig. S7D), indicating functional engagement of STAT5 signaling.

**Fig. 4.**
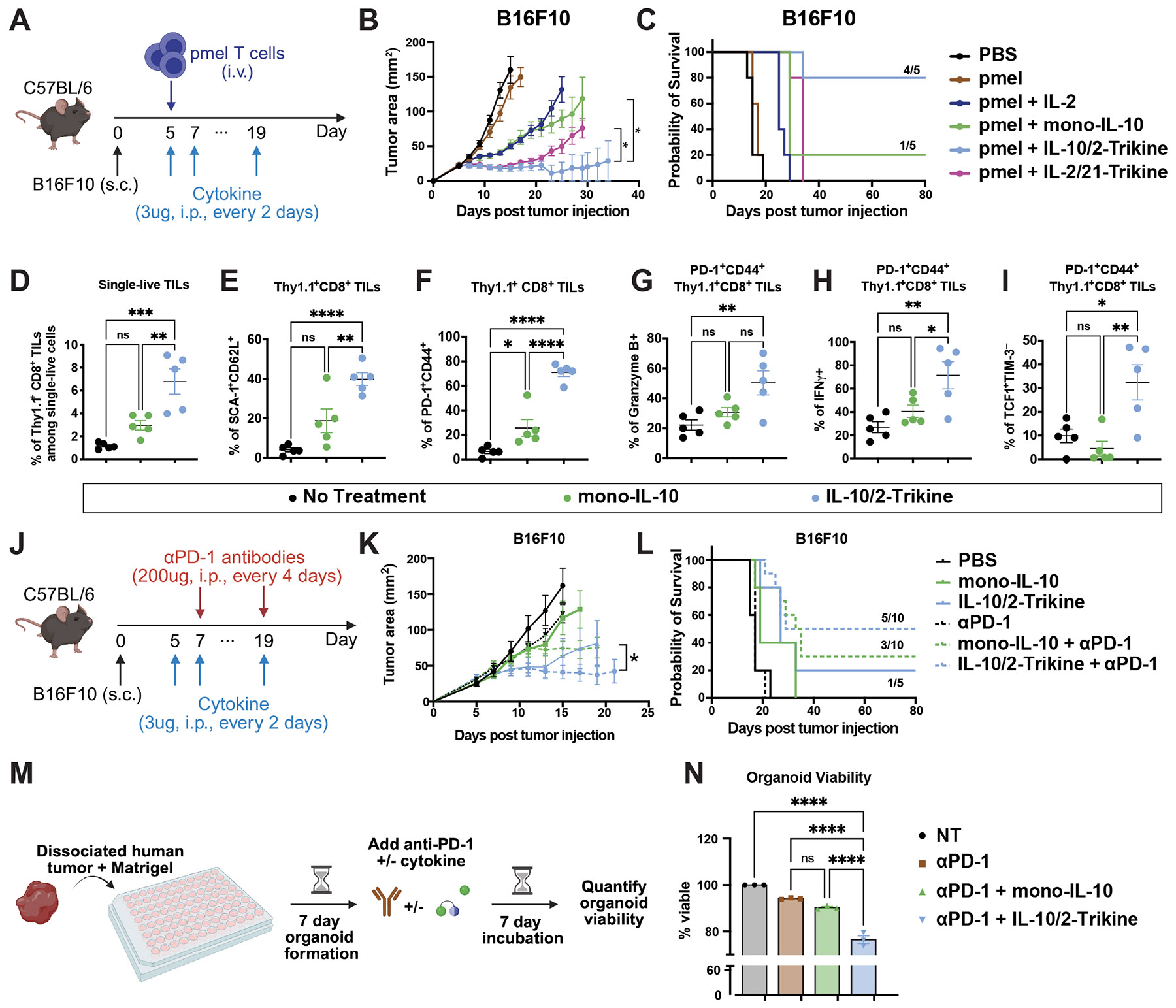
IL-10/2-Trikine preserves stem-like, antigen-experienced TILs and enhances ICB efficacy in melanoma. (**A-C**), C57BL/6 mice bearing established s.c. B16F10 tumors received intravenous (i.v.) adoptive cell transfer (ACT) of pmel CD8^+^ T cells (5 × 10^6^) on day 5, followed by i.p. injections of cytokine (3 μg functional cytokine) or PBS every other day until day 20 (n = 5 animals); the experimental timeline (A), average tumor growth curves (B), and survival curves (C). Results representative of 2 independent experiments. (**D-I**) Experimental setting is described in Fig. S6F. C57BL/6 mice bearing established s.c. B16F10 tumors received i.v. ACT of pmel CD8^+^ T cells (5 × 10^6^) on day 9, followed by i.p. injections of cytokine (3 μg functional cytokine) or left NT on days 9 and 11 (n = 5 animals). Mice were sacrificed on day 13, tumors were collected for flow cytometry analysis. Frequencies of Thy1.1^+^CD8^+^ tumor-infiltrating lymphocytes (TILs) among single-live cells (D), frequencies of SCA-1^+^CD62L^+^ cells among Thy1.1^+^CD8^+^ TILs (E), frequencies of PD-1+CD44+ cells among Thy1.1+CD8+ TILs (F) frequencies of Granzyme B+ cells among PD-1+CD44+ Thy1.1+CD8+ TILs (G), frequencies of IFN-γ+ cells among PD-1+CD44+ Thy1.1+CD8+ TILs (H), frequencies of TCF1+TIM-3- cells among PD-1+CD44+ Thy1.1+CD8+ TILs (I). Results representative of 2 independent experiments. (**J-L**), C57BL/6 mice bearing established s.c. B16F10 tumors (n = 5-10 animals) were administered cytokine (3 μg functional cytokine), PBS, or left NT i.p. on day 5 and every other day until day 19. For combination therapy, anti-PD-1 antibody (200 μg per dose) were i.p. administered on days 7, 11, 15, and 19; the experimental timeline (J), average tumor growth curves (K), survival curves (L). Results representative of 2 independent experiments. (**M**) Schematic of patient-derived organoid experiment. (**N**) Percentages of T133 tumor organoid viability in experiment as in (M). Results representative of 2 independent experiments. All data represent mean ± s.e.m. and are analyzed by one-way (D-I, N) or two-way (B, K) ANOVA with Tukey’s post-test. Schematics in D and M created using BioRender.com.

We performed a dose-response efficacy and toxicity assessment, concluding that 3 μg of IL-10/2-Trikine was well tolerated and was the minimum dose needed to see maximal effect (Fig. S7E-F). To interrogate the impact of the distinct STAT3/STAT5 signaling, we first turned to adoptive T cell transfer models of solid tumors. C57BL/6 mice bearing subcutaneous B16F10 tumors received Thy 1.1+ pmel CD8+ T cells via ACT followed by treatment with IL-2, mono-IL-10 and the IL-10/2-Trikine (Fig. 4A). Notably, the IL-10/2-Trikine demonstrated superior therapeutic efficacy relative to IL-2, mono-IL-10, and the IL-2/21-Trikine, achieving long-term tumor control in 80% of mice (Fig. 4B-C, S7G). In addition, the IL-10/2-Trikine significantly expanded transferred Thy1.1+CD8+ T cells in the spleen and tumor relative to mono-IL-10 or non-treated (NT) controls (Fig. 4D, S7H-I). We also observed increased expression of anti-apoptotic proteins Bcl-2 and Bcl-6 in Thy1.1+CD8+ TILs post IL-10/2-Trikine treatment (Fig. S7J-K) (44). Consistent with *in vitro* findings, the IL-10/2-Trikine markedly enriched SCA-1+CD62L+ cells among Thy1.1+CD8+ TILs (Fig. 4E). These SCA-1+CD62L+ cells represent PD-1+CD44+ antigen-experienced TILs with stem-like properties (Fig. 4F, S8A-B) (45). Within the PD-1+CD44+ compartment, IL-10/2-Trikine treatment significantly enhanced granzyme B and IFN-γ production (Fig. 4G-H). Further phenotyping using TCF1 and TIM-3 revealed that the PD-1+CD44+ TILs expanded by the IL-10/2-Trikine were predominantly TCF1+TIM-3-, a subset previously associated with responsiveness to PD-1 blockade (Fig. 4I) (46).

Building on this observation, we investigated whether the IL-10/2-Trikine could potentiate PD-1 blockade in the absence of pmel ACT. In the B16F10 melanoma model, which is largely resistant to PD-1 monotherapy (47), the combination of the IL-10/2-Trikine and PD-1 blockade markedly improved therapeutic outcome, resulting in complete tumor regression and long-term survival in 50% of treated mice (5/10) (Fig. 4J-L, S8C), and all treatments were well tolerated, with no significant weight loss observed (Fig. S8D). This enhanced efficacy was accompanied by an increased frequency of gp100 tetramer^+^ T cells in only the IL-10/2-Trikine + anti-PD-1 treatment group (Fig. S8E-F). By contrast, mono-IL-10 also demonstrated synergy with PD-1 blockade, albeit via a distinct mechanism and with reduced efficacy compared to the IL-10/2-Trikine, achieving tumor clearance in 3 out 10 of mice (Fig. 4L).

To extend these findings to a human setting, we evaluated the IL-10/2-Trikine in the PD-1 blockade refractory patient (T133)-derived tumor organoid (Fig. 4M) (48). In this model, the IL-10/2-Trikine combined with nivolumab (anti-PD-1) significantly enhanced tumor cell killing, reducing organoid viability to 76.4% versus 94.1% with nivolumab alone (Fig. 4N). TILs treated with the IL-10/2-Trikine secreted 3.6- to 4.8-fold higher levels of IL-4, IFN-γ, and granzyme A compared to NT controls (Fig. S8G). In parallel, autologous peripheral blood T cells showed increased responsiveness to PD-1 blockade in the presence of IL-10-trikine, exhibiting enhanced tumor cytotoxicity and elevated granzyme B and IFN-γ secretion relative to mono-IL-10 (Fig. S8H-J).

## IL-10/2-Trikine unleashes TIL responses to PD-1 blockade in SCLC

**Fig. 5.**
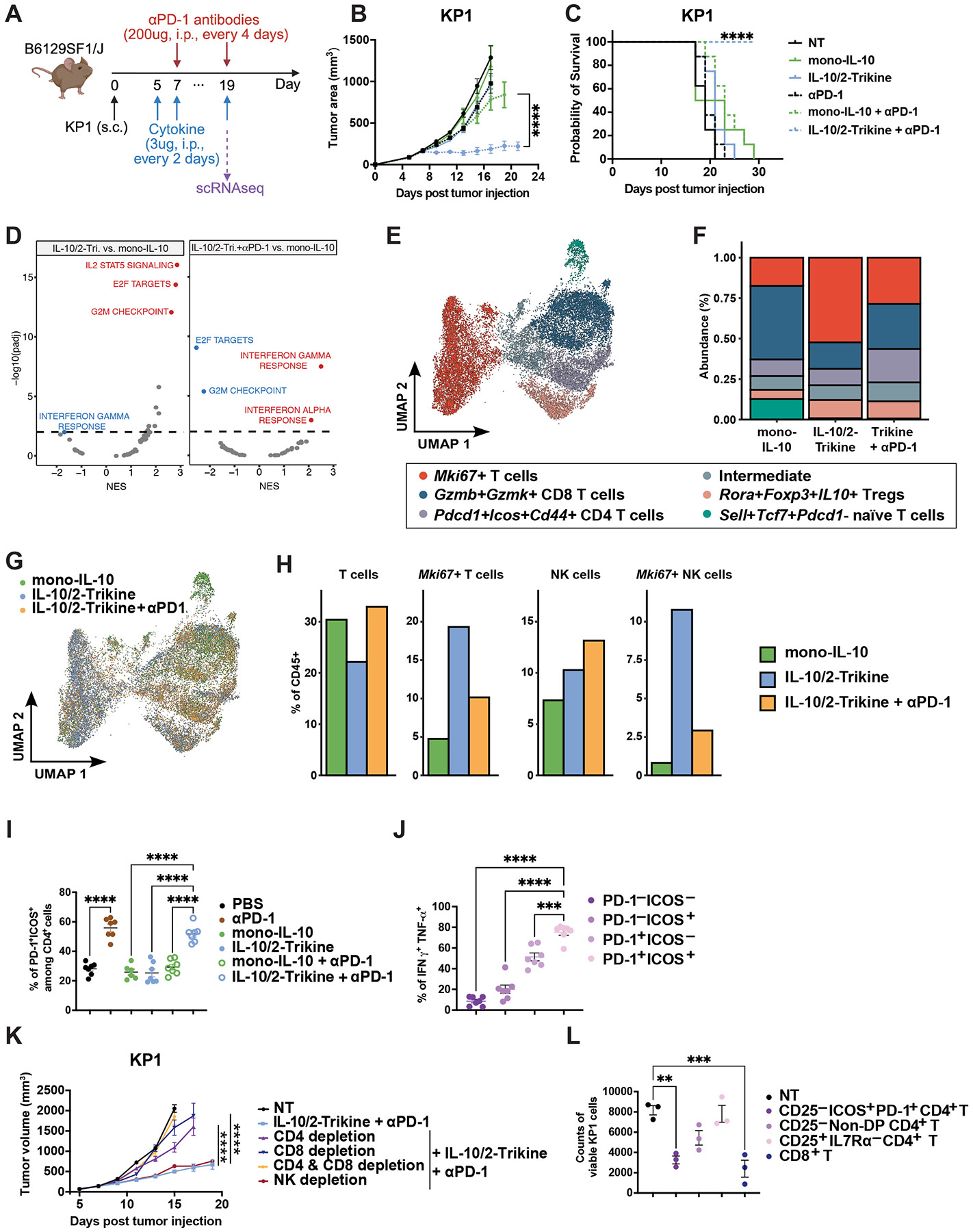
IL-10/2-Trikine sensitizes SCLC to PD-1 blockade. (**A-C**), B6129SF1/J (F1) mice (n = 8 animals) bearing established s.c. KP1 tumors were administered cytokine (3 μg functional cytokine), PBS, or left NT i.p. on day 5 and every other day until day 19. For combination therapy, anti-PD-1 antibody (200 μg per dose) was i.p. administered on days 7, 11, 15, and 19; the experimental timeline (A), average tumor growth curves (B), and survival curves (C). Statistical significance in (C) was calculated using a Mantel-Cox test, with **** representing the statistical significance (p < 0.0001) of the IL-10/2-Trikine + αPD-1 survival curve over those of NT, IL-10/2-Trikine, and αPD-1. Results representative of 2 independent experiments. (**D-H**), Experimental setup is described in Fig. 5A. Mice were sacrificed on day 19, and CD45^+^ TILs were sorted for scRNA-seq analysis. Gene Set Enrichment Analysis (GSEA) for differentially expressed genes (DEGs) between T cells from IL-10/2-Trikine and mono-IL-10 treatment groups using Hallmark gene sets (left); GSEA for DEGs between T cells from IL-10/2-Trikine + anti-PD-1 and IL-10/2-Trikine treatment groups using Hallmark gene sets (right) (D). Merged UMAP of unsupervised cell clusters (E). Cluster composition for each treatment group (F). Distribution of each treatment group across the UMAP space (G). Relative abundance of T, *Mki67+* T, NK, and *Mki67+* NK cells among CD45+ cells by treatment group (H). scRNAseq data are the result of one independent experiment. (**I-J**), F1 mice bearing KP1 tumors were administered i.p. mono-IL-10, IL-10/2-Trikine (3 μg functional cytokine per dose), or PBS every other day from day 7 to day 17. Mice also received i.p. injections of anti-PD-1 antibody (200 μg per dose) on days 9, 13, and 17. On day 18, mice were sacrificed, and tumors were collected for flow cytometry analysis (n = 7 animals). Frequencies of PD-1^+^ICOS^+^ cells among CD4^+^ TILs (I). Frequencies of IFN-γ+TNF-α+ cells among PD-1/ICOS quadrant-gated subsets (J). Results representative of 2 independent experiments. (**K**), B6129SF1/J mice bearing KP1 tumors were treated intraperitoneally with IL-10/2-Trikine (3 μg functional cytokine per dose) every other day from day 7 to day 17. Anti–PD-1 antibody (200 μg per dose) was administered i.p. on days 9, 13, and 17. Depleting antibodies (400 μg per dose) were administered i.p. on days 6, 9, and 13 (n = 6 animals). Shown are the average tumor growth curves. (**L**), Counts of viable KP1 tumor cells. Results representative of 2 independent experiments. All data represent mean ± s.e.m. and are analyzed by one-way (I, J, L) or two-way (B, K) ANOVA with Tukey’s post-test. Schematic in A created using BioRender.com.

Small-cell neuroendocrine tumors, including small-cell lung cancer (SCLC), are aggressive and often characterized by low major histocompatibility class I (MHC-I) expression and poor response to current therapies (49-51). We investigated the therapeutic potential of the IL-10/2-Trikine combined with PD-1 blockade in the *Rb/p53* mutant KP1 SCLC model (Fig. 5A) (52). KP1 tumors exhibited resistance to all monotherapies, including cytokines and PD-1 blockade alone, as well as PD-1 combined with mono-IL-10 (Fig. 5B-C, S9A). Remarkably, only the combination of the IL-10/2-Trikine with anti-PD-1 therapy elicited profound tumor suppression. All treated mice demonstrated durable survival benefits, surviving up to 30 days post-tumor inoculation without observable toxicity (Fig. 5C, S9B).

We performed single-cell RNA sequencing of CD45+ TILs (Fig. 5A). Gene set enrichment analysis revealed a significantly enhanced IL-2-driven STAT5 signaling gene signature with the IL-10/2-Trikine, indicating functional IL2Rβ-mediated STAT5 activation (Fig. 5D). Compared to the IL-10/2-Trikine alone, the combination with PD-1 blockade enriched interferon response gene signatures (Fig. 5D). Unsupervised clustering of T cells identified six transcriptionally distinct subsets (Fig. 5E-F, S9C): cycling *Mki67*+ T cells, *Gzmb*+*Gzmk*+ CD8 T cells, *Pdcd1*+*Icos*+*Cd44*+ CD4 T cells (Fig. S9D), an intermediate cluster bridging the *Mki67*+ T cell cluster and others, *Rora*+*Foxp3*+*IL10*+ Tregs, and *Sell*+*Tcf7*+*Pdcd1*- naïve T cells. Notably, the IL-10/2-Trikine significantly expanded the cycling *Mki67*+ T cell and NK cell clusters compared to mono-IL-10, accompanied by upregulation of cell cycle genes (*Cdk1*, *Cdk2*, *Cdk4*, *Cdk7*, *Cdk9*, *Mki67*) (Fig. 5F-H, S10A-B). Consistently, two doses of IL-10/2-Trikine treatment increased BrdU incorporation and Ki-67 expression in CD4+ and CD8+ TILs (Fig. S10C-I). Compared to the IL-10/2-Trikine alone, the addition of PD-1 blockade reduced the cycling *Mki67*+ T cell cluster while expanding the *Gzmb*+*Gzmk*+ CD8 T cells cluster and *Pdcd1*+*Icos*+*Cd44*+ CD4 T cell cluster, suggesting that checkpoint inhibition facilitates the transition of cycling cells into activated states (Fig. 5F-G). Among all clusters, only the *Pdcd1*+*Icos*+*Cd44*+ CD4 T cell cluster was selectively enriched by the combination therapy relative to both the IL-10/2-Trikine and mono-IL-10. This subset uniquely co-expressed high levels of *Pdcd1*, *Cd44*, and *Icos*, and exhibited elevated *Ifng* expression compared to other T cell clusters (Fig. S9D), and we further examined this population of TILs via flow cytometry.

Flow cytometry demonstrated that the IL-10/2-Trikine plus PD-1 blockade elicited the most robust expansion of CD45+ TILs, with a preferential increase in CD4+ T cell density and a trend toward enhanced CD8+ infiltration (Fig S11A-D). This bias is conceivably linked to the CD4+-skewed immune landscape of KP1 tumors and preferential expression of IL-10R and IL2Rβ on CD4+ T cells (Fig. S11C-F). Among CD4+ TILs, PD-1 blockade promoted an effector phenotype, marked by increased frequencies of PD-1+ICOS+ double-positive (DP) cells with elevated CD44 expression (Fig. 5I, S11G) (53). Given that PD-1 and CD44 are co-expressed in the TILs of KP1 tumors, we focused on PD-1+ICOS+ cells and excluded CD44 from further gating (Fig. S11H-I). Compared to non-DP subsets, PD-1+ICOS+ CD4+ T cells produced significantly higher levels of IFN-γ and TNF-α and expressed elevated CXCR6 (Fig. 5J, S11J).

To assess whether these DP CD4+ T cells contribute to the therapeutic efficacy of combination therapy, we performed immune subset depletion. Ablation of CD4+ T cells (including Tregs) or CD8+ T cells, but not NK cells, nearly completely abrogated tumor control, indicating that both CD4+ and CD8+ T cells are essential for antitumor responses (Fig. 5K). We next examined whether PD-1+ICOS+ CD4+ TILs possess direct tumor killing capacity. From KP1 tumors treated with the IL-10/2-Trikine + PD-1 blockade, we isolated CD25-PD-1+ICOS+ CD4+ T cells, CD25- non-DP CD4+ T cells, CD25+IL-7Rα- Tregs, and CD8+ T cells, and cocultured them with KP1 tumor cells. While CD8^+^ T cells remained the dominant cytotoxic subset, PD-1+ICOS+ CD4+ T cells exhibited robust tumor killing activity, comparable to CD8+ T cells (Fig. 5L). Functionally, these DP CD4+ T cells secreted high levels of IL-17A and IL-22, along with modestly elevated IFN-γ, IL-6, IL-4, and IL-10 (Fig. S11K). Together, these findings indicate that the IL-10/2-Trikine markedly enhances the efficacy of PD-1 blockade in SCLC, with tumor control mediated by both PD-1+ICOS+ CD4+ T cells and CD8+ TILs.

## IL-10/2-Trikine monotherapy boosts anti-tumor immunity in melanoma and pancreatic cancer

**Fig. 6.**
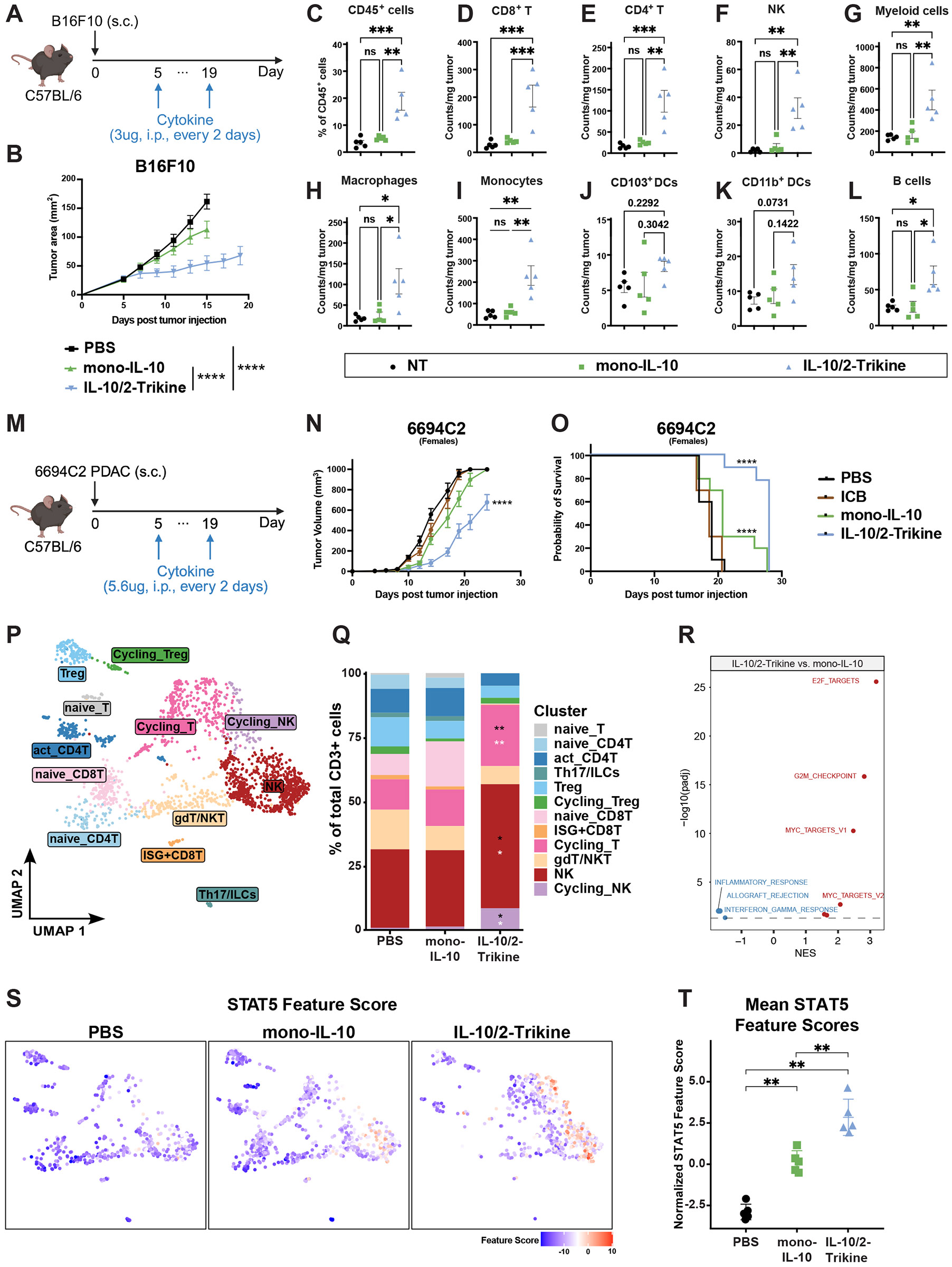
IL-10/2-Trikine monotherapy suppresses poorly immunogenic tumors in murine melanoma and pancreatic cancer models. (**A-B**), C57BL/6 mice bearing established s.c. B16F10 tumors received i.p. injections of PBS, mono-IL-10, or IL-10/2-trikine (equivalent to 3 μg functional IL-10) starting on day 5, administered every other day until day 19 (n = 9 animals); the experimental timeline (A) and average tumor growth curves (B). Results are combined from 2 independent experiments. (**C-L**), C57BL/6 mice bearing established s.c. B16F10 tumors received i.p. injections of cytokine (3 μg functional cytokine) or left NT on days 9 and 11 (n = 5 animals). Mice were sacrificed on day 13, tumors were collected for flow cytometry analysis. (C), Average frequencies of CD45^+^ cells among single-live cells. (D- F), Counts of CD8^+^ T cells (D), CD4^+^ T cells (E), NK cells (F), myeloid cells (G), macrophages (H), monocytes (I), CD103^+^ dendritic cells (J), CD11b^+^ dendritic cells (K), and B cells (L) per mg of tumor tissue. Results are representative of 2 independent experiments. (**M-O**), C57BL/6 mice bearing established subcutaneous C2 pancreatic tumors were treated intraperitoneally with cytokine (5.6 μg functional cytokine per dose) or left untreated (NT), administered every other day from day 6 to day 20 (n = 5 mice per group); experimental timeline (M), average tumor growth curves (N) and survival curves (O) of female mice in experiment described in (M). Results are representative of 2 independent experiments. Indicated statistical significance is IL-10/2-Trikine vs. PBS, IL-10/2-Trikine vs. ICB, and IL-10/2-Trikine vs. mono-IL-10. Indicated statistical significance in (O) is mono-IL-10 vs. PBS, is IL-10/2-Trikine vs. PBS, IL-10/2-Trikine vs. ICB, and IL-10/2-Trikine vs. mono-IL-10. Data represent mean ± s.e.m. and are analyzed by one-way (C to L) or two-way (B, N) ANOVA with Tukey’s post-test. Schematics in A and M created using BioRender.com. (**P-T**) C57BL/6 mice were inoculated subcutaneously. with 2.5 × 10^5^ 6694c2vTRP1 cells and treated with PBS, mono-IL-10 (26 μg/mouse, n = 5), or IL-10/2-Trikine (30.6 μg/mouse, n = 5) intraperitoneally on days 8 and 10 post-inoculation. Tumors were harvested on day 14 and CD45+ cells were isolated by magnetic bead separation, individually labeled with hashtag antibodies, pooled by treatment group, and subjected to Single Cell 5' RNA and cell surface protein sequencing. (P) UMAP of tumor-infiltrating CD3^+^ T and NK cells colored by unsupervised clusters. (Q) Bar graph shows cell cluster composition across treatment groups. Statistical significance was assessed by unpaired t test. Black asterisks indicate comparisons between PBS and IL-10/2-Trikine; white asterisks indicate comparisons between IL-10 and IL-10/2-Trikine. *P < 0.05, **P < 0.01. (R) Gene Set Enrichment Analysis (GSEA) of differentially expressed genes (DEGs) between all clusters from IL-10/2-Trikine–treated and mono–IL-10–treated groups using MSigDB Hallmark gene sets. (S) UMAP of tumor-infiltrating T/NK cells colored by a STAT5 target gene expression score (*Gzmb*, *Prf1*, *Nkg7*, *Gzma*, *Klrd1*, *Klrb1c*, *Klra7*, *Ccr5*, *Ly6a*, *Thy1*). (T) Mean STAT5 scores per mouse across all clusters, normalized per mouse. Each dot represents one mouse; error bars indicate standard deviation. Statistical significance was assessed by Wilcoxon test. **P < 0.01. scRNAseq data are the result of one independent experiment.

We next evaluated the therapeutic potential of the IL-10/20-Trikine as a monotherapy. In the B16F10 melanoma model, IL-10/2-Trikine monotherapy induced complete tumor regression in 2 of 9 mice, whereas mono-IL-10 failed to elicit curative responses (Fig. 6A-B, Fig. S12A-B). The observed variability in therapeutic outcomes among mice treated with the IL-10/2-Trikine may be attributed to differences in pre-existing T cell clonality (Fig. S12A). Immune profiling revealed robust activation following two doses of treatment, with CD45+ tumor-infiltrating immune cells increasing from 3.52% (NT) to 18.86% among live singlets (Fig. 6C). The densities of all major immune subsets were broadly elevated (Fig. 6D-L).

Encouraged by these findings, we evaluated the IL-10/2-Trikine in the 6694C2 KPC pancreatic ductal adenocarcinoma (PDAC) model, which is minimally infiltrated by T cells and refractory to all standard immunotherapies, including combination immune checkpoint blockade (ICB) (Fig. 6M). While immune checkpoint blockade and chimeric antigen receptor T-cell therapies have revolutionized the treatment of many cancers, they have not proved effective in the highly immunosuppressive tumor microenvironment of human PDAC, and this finding is recapitulated in the 6694C2 KPC model (54, 55). Pegylated IL-10 reached a phase III clinical trial for PDAC but ultimately demonstrated only limited clinical efficacy (20, 56, 57). In contrast, the IL-10/2-Trikine substantially suppressed tumor progression in the subcutaneous 6694C2 PDAC model without toxicity in both male and female mice, whereas mono-IL-10 showed no therapeutic benefit (Fig. 6M-O, Fig. S12D-L).

To better understand the unique contribution of IL-10/2-Trikine, we performed single cell transcriptional profiling of mid-stage tumors, prior to divergence in size (Fig. 6P, S13A). Similar to the results in our KP.1 model, the IL-10/2-Trikine induced proliferation of both T and NK cells even in the absence of PD-1 blockade, with significant increases in cycling populations of immune cells, and total NK cells compared to PBS or mono-IL-10 treatment alone (Fig. 6Q). Mono-IL-10 treatment alone failed to increase immune cell proliferation or cytotoxic signatures in the tumor infiltrates (Fig. S13B-E). Upregulation of STAT5 target genes was also evident at the gene expression level and in gene set enrichment for cell cycle genes, validating the design goal (Fig. 6R-T).

The efficacy and tolerability of the IL-10/2-Trikine, as monotherapy or in combination with anti-PD-1, across refractory tumor models demonstrates its powerful ability to promote immune cell infiltration (Fig. 3-6, S6-12). Moreover, its species cross-reactivity combined with promising data on primary patient tumor samples bode well for clinical translation (Fig. 4, S8G-I).

## Discussion

Trikines provide a cytokine engineering strategy for generating custom-designed pSTAT signaling profiles without genetic modification of target cells. By compelling assembly of a three-chain receptor complex in *cis*, Trikines enable AND-gated signaling that is restricted to cells co-expressing two cytokine receptors, while a non-agonist binding module recruits a third receptor chain to modulate downstream STAT activation. This design principle allows cytokine receptor subunits to be effectively “mixed and matched,” creating signaling outputs that are not induced by natural cytokines. Importantly, the magnitude and identity of the acquired STAT signal can be tuned through the third chain, enabling controlled perturbation of cellular state. Trikines are not defined by a single optimal molecule, but by a design principle—controlled rebalancing of STAT signaling atop a dominant cytokine scaffold. In this framework, individual Trikines represent complementary implementations tailored to distinct biological and translational contexts.

Consistent with this design logic, Trikines produced both anticipated and emergent biological effects. For the IL-2/21 Trikine, incorporation of a STAT3 signal into an IL-2–dominant context restrained terminal differentiation and exhaustion while preserving antitumor efficacy, as expected based on prior work linking STAT3-associated programs to stem-like T cell states. Unexpectedly, this configuration also substantially reduced IL-2–associated toxicity, driven by attenuated activation of NK cells. Conversely, the IL-10/2-Trikine was designed to introduce STAT5 signaling into a STAT3-dominant cytokine scaffold, thereby enhancing proliferation of activated lymphocytes. While this goal was achieved, the extent of immune infiltration and efficacy in poorly immunogenic tumors was unanticipated. In our lung cancer model, tumor control was observed only in combination with PD-1 blockade and showed a cooperative contribution of CD4+ and CD8+ T cells, which represents an important area for future investigation. In poorly immunogenic pancreatic cancer, we observed monotherapy efficacy of IL-10/2-Trikine potentially driven by a combination of proliferating T and NK cells even in the absence of PD-1 blockade. In both models, the addition of STAT5 signaling to sustain robust accumulation of tumor-infiltrating lymphocytes appears critical.

Previous efforts to engineer tandem cytokines have largely relied on fusion of two fully active agonists, a strategy that retains the pleiotropy and toxicity of both components and has limited clinical translation (58-60). More recently, targeted cytokine fusions such as EGFR-directed IL-2–IL-10 constructs have been explored to locally modulate immune responses within the TME (61). In contrast, Trikines are not dual agonists but AND-gated agonists, in which a non-agonist third arm enforces cis assembly of a ternary receptor complex on a single cell. This architecture minimizes systemic pleiotropy and avoids the liabilities associated with simultaneous activation of two independent cytokine pathways. Consistent with this mechanism, transcriptomic analyses reveal that Trikines induce emergent gene expression programs that are distinct from those elicited by simply combining their parent cytokines.

Viewed more broadly, Trikines constitute a modular discovery platform for therapeutic cytokines. This approach requires only knowledge of receptor expression patterns and does not depend on gene delivery or cell engineering. By starting from a cytokine scaffold with established biology and systematically introducing defined STAT perturbations, Trikines enable exploration of nearby functional space rather than an unconstrained search for novel activities. This framework provides a generalizable strategy for reprogramming cytokine signaling with precision, opening new avenues for immunotherapy design.

## Materials and Methods:

### Cells, cell lines, and cell culture

YT-1 cells (62) and YT-1 γ_c_ KO cells were cultured in RPMI medium(Gibco) supplemented with 10% FBS, penicillin streptomycin, 10 mM HEPES, 1x MEM Non-Essential Amino Acids (Gibco), and 1x Glutamax (Gibco). B16F10 cells (ATCC) were cultured in DMEM medium (Gibco) supplemented with 10% FBS and penicillin streptomycin. MC38 cells (Sigma) were cultured in DMEM medium supplemented with 10% FBS, penicillin streptomycin, 1 mM sodium pyruvate, 1x MEM Non-Essecntial Amino Acids, and 1x Glutamax. CT26 (ATCC) cells were cultured in RPMI medium supplemented with 10% FBS and penicillin streptomycin. Human melanoma cell line nRFP+ M407 cell line was established, developed, and cultured as previously described (8). Rb/p53 mutant mouse small cell lung cancer (SCLC) KP1 cells were previously described and propagated in the lab (SCLC-A subtype) (52). KP1 cells were cultured in RPMI-1640 supplemented with fetal bovine serum (FBS) (10% v/v, Hyclone), 1× GlutaMax (Gibco/Thermo Fisher Scientific), and penicillin/streptomycin (1% v/v, Gibco/Thermo Fisher Scientific). 6694C2 cells were a gift from Dr. Ben Stanger (University of Pennsylvania) (54). 6694C2 cells were cultured in DMEM medium supplemented with 10% FBS and 2 mM glutamine.

B16F10 tumor cells (4 × 10^5^) were implanted subcutaneously (s.c.) into the right flanks of female C57BL/6 wild-type (WT) mice to establish the subcutaneous melanoma model. KP1 tumor cells (5 × 10^5^) were inoculated s.c. into the right flanks of female B6129SF1/J mice to establish the subcutaneous SCLC model. 6694C2 tumor cells or 6694c2vTRP1 tumor cells (2.5 x 10^5^) were inoculated s.c. into the right flanks of male and female C57BL/6 mice to establish the subcutaneous PDAC model.

For experiments performed at Stanford University, human PBMCs were isolated from LRS chambers (Stanford Blood Center, check Wherry and Ribas) and cryopreserved until time of use. For experiments performed at the University of Pennsylvania, healthy donor PBMCs were obtained by the University of Pennsylvania Human Immunology Core. PBMCs were purified from whole blood or leukapheresis products by Ficoll-Hypaque density gradient centrifugation. Donors were self-identified as healthy.

Mouse splenocytes and lymphocytes were isolated from the spleen and lymph nodes of female C57BL/6 mice. T cells were prepared from thawed PBMCs (human) or freshly isolated splenocytes and lymphocytes (mouse) by 3-4 day activation by plate-bound aCD3, soluble aCD28, with or without 100 IU/mL MSA-tagged human or mouse IL-2 in the medium unless otherwise indicated. Cells were rested 18-24 hours in media lacking serum, cytokines, or activating antibodies prior to signaling assays. T cell culture medium was comprised of RPMI medium supplemented with 10% FBS, 1x GlutaMAX, 1x Penicillin streptomycin, NEAA, Sodium Pyruvate, 10 mM HEPES, and 20 mM 2-Mercaptoethanol (Sigma M3148). NY-ESO transduced T cells were generated from PBMCs as described previously (8).

Expi293 suspension cells (Thermo Fisher) were cultured in Expi293 Expression Medium on a 120rpm shaking platform. All cell cultures were maintained at 37C in a 5% CO2 humified incubator.

For *ex vivo* processing of mouse cells, the spleen, right inguinal lymph node, and tumor (of tumor bearing mice) were harvested at the time of necropsy. Organs were washed with PBS and pushed through a 70 μm cell strainer. Splenocytes were treated with ACK lysis buffer before subsequent procedures. Tumors were minced, washed with PBS, and pushed through a 70 μm cell strainer.

### Protein production and purification

The cDNAs encoding recombinant proteins was cloned into the mammalian expression vector pD649 (ATUM DNA 2.0), which includes an HA secretion signal peptide and a C-terminal 8×His tag for affinity purification. DNA encoding was purchased from Integrated DNA Technologies (IDT) and cloned into pD649 as an N-terminal fusion. Mammalian expression DNA constructs were transfected into HEK293F cells using the Expi293^™^ Expression System (BD Biosciences) for secretion and purified from the clarified supernatant by nickel affinity resin (Ni-NTA, Qiagen) followed by size-exclusion chromatography with a Superdex-200 column (Cytiva) and formulated in sterile phosphate-buffered saline (PBS) for injection. Endotoxin was removed using the Proteus NoEndo HC Spin column kit following the manufacturer’s recommendations (VivaProducts) and endotoxin removal was confirmed using the Pierce LAL Chromogenic Endotoxin Quantification Kit (Thermo Fisher Scientific). Proteins were concentrated, flash frozen in liquid nitrogen and stored at −80 °C until ready for use.

The recombinant proteins used in the *in vitro* experiments showed in Figures 1B, 1D, 1E, 1G-I, S1C-D, and S1F-G were produced without an mouse serum albumin (MSA) tag. MSA-tagged proteins were used in all other experiments, *in vitro* and *in vivo.*

### Activation and expansion of primary human T cells

Primary human peripheral blood mononuclear cells (PBMCs) isolated from the melanoma patient donors were thawed and resuspended at a cell density of 2 × 10^6^ cells/ml in T cell medium supplemented with MSA-human-IL-2 (MSA-hIL-2) (200 U ml^−1^). For human T cell activation, cells were activated with plate-bound anti-human CD3ε (1 μg ml^−1^, clone OKT-3, BioXCell) and soluble anti-human CD28 (5 μg ml^−1^, clone 9.3, BioXCell). After 48 hours of activation, T cells were collected and expanded in T cell medium supplemented with MSA-hIL-2 (200 U ml^−1^). The medium was changed every other day. Cells were harvested for in vitro assays 8–15 days after activation.

### Generation of YT-1 cell line with γ_c_ knockout

To knockout γc in YT-1 cells, we used a lentiviral CRISPR/Cas9 system following the GeCKO protocol (63). Two sgRNA sequences (5'-AACGCTACACGTTTCGTGTT-3' and 5'-TCGAGTACATGAATTGCACT-3') were cloned into lentiCRISPR-v2 plasmids (Addgene, 98290), transformed into Stbl3 competent E. coli cells, and purified using standard plasmid preparation methods. HEK293T cells were seeded at 6 × 10^5 cells per well in 6-well plates in DMEM supplemented with 10% FBS, 2 mM GlutaMAX, and antibiotics. After 24 hours of incubation at 37°C and 5% CO2, cells were transfected using Fugene HD (Promega) in Opti-MEM (Gibco) according to the manufacturer's instructions. Transfections included 700 ng of lentiCRISPR-v2 plasmid containing the target sgRNAs, 500 ng of packaging plasmid psPAX2 (Addgene, 12260), and 260 ng of envelope plasmid pMD2.G (Addgene, 12259). Forty-eight hours post-transfection, viral supernatants were collected, centrifuged at 4,000 rpm for 5 minutes to remove debris, and used to transduce YT-1 cells at a density of 1 × 10^6 cells in RPMI 1640-GlutaMAX medium containing 10% FBS, nonessential amino acids, 15 mM HEPES, and antibiotics. Transduced YT- cells were enriched and selected using magnetic activated cell sorting (MACS) and fluorescence-activated cell sorting (FACS). The knockout efficiency and surface expression of γ_c_ were assessed by flow cytometry using fluorescently labeled antibodies.

### pSTAT signaling assays

pSTAT signaling assays were performed on unlabeled cells or cells pre-labeled with fluorescent antibodies targeting surface markers. Ligands were added to cells at indicated concentrations and incubated in serum-free media at 37C for 15-20 minutes, followed by fixation with 1.5% paraformaldehyde. Cells were washed and then permeabilized with ice-cold methanol at −80C. The cells were then washed and stained with fluorescent pSTAT detection antibodies for 2 hours, followed by analysis via flow cytometry on a Beckman-Coulter Cytoflex-S.

### Anti-human IL-21Rα VHH selection

IL-21Rα ectodomain was cloned with an AviTag tag into the pD649 vector and expressed in Expi293 cells as previously described. IL-21Rα was biotinylated with BirA and purified by size exclusion chromatography. Nanobodies with affinity for IL-21Rα were selected from a nanobody yeast display library as previously described (40). 5 rounds of magnetic activated cell sorting (MACS) were followed by 2 rounds of fluorescence-activated cell sorting (FACS). In the final round of selection, yeast were stained with 10nM IL-21Rα overnight, followed by secondary staining with anti-HA AlexaFluor488 and streptavidin-AlexaFluor647 (SA-647) for 30 minutes. Single clones were sorted by fluorescence-activated cell sorting (Sony SH800) and sequenced by PCR. The top nine unique nanobodies were then recombinantly cloned into pD649 vector with an AviTag and expressed in Expi293 cells. Nanobodies were biotinylated with BirA and purified by size exclusion chromatography. Staining of endogenous IL-21Rα on human immune cells was validated by staining YT-1 cells a titration of nanobody for 2 hours, followed by secondary staining with streptavidin-647 for 30 minutes. Fluorescence was analyzed by flow cytometry. Nanobody GA179 was found to stain YT-1 cells most robustly in a dose-dependent manner and was selected for downstream study.

### Surface plasmon resonance (SPR)

SPR binding studies were performed using a Biacore T100 (Cytiva) operating on Biacore T100 control software version 2.0.4. Approximately 210 RU of site-specifically biotinylated human IL-21Rα (SinoBiological) or unrelated negative-control reference protein were immobilized onto a Series S Sensor Chip (Cytiva). For each binding cycle, a regeneration condition of 10 mM Glycine-HCl, pH 3.0 was used. Data was processed using Biacore T100 Evauation Software, version 2.0.4 (Cytiva). Dissociation constants were calculated using kinetic fit analysis.

### *In vitro* cytokine production assays

For experiments performed at Stanford University, pre-activated human or mouse T cells were cultured with cytokines for indicated time frames. For *ex vivo* assays, mouse lymphocytes were isolated from the lymph nodes, spleen, and tumor from individual mice. Cells were incubated with Cell Stimulation Cocktail (plus protein transport inhibitors) (eBioscience) for 4-6 hours, stained for indicated cell surface markers, followed by fixation and permeabilization via BD Cytofix/Cytoperm Fixation/Permeabilization Kit. The cells were then stained for indicated cytokines and fluorescence was detected and analyzed via a Beckman-Coulter Cytoflex-S and FlowJo software, respectively.

For experiments performed at the University of Pennsylvania, T cells were taken from the *in vitro* exhaustion assay and resuspended at 1x10^7^ T cells/ml. 100 μL of T cells were plated in 96-well flat bottom tissue culture plates and cultured in the presence of NALM6 at + Protein Transport Inhibitor Cocktail at 37C for 5 hours. Staining was performed as previously mentioned. eBioscience^™^ Foxp3 / Transcription Factor Staining Buffer Set was used for intracellular staining.

### Flow cytometry analyses

For experiments performed at Stanford University, cells were collected into U-bottom 96-well plates (Thermal Fischer Scientific), blocked with anti-mouse CD16/32 antibody (BioLegend) or human TruStain FcX^™^ (Biolegend), and incubated with indicated surface marker staining antibodies at 4 °C for 20 min, followed by live/dead staining by 4',6-diamidino-2-phenylindole (DAPI, Thermo Fisher Scientific). Cells were then washed and resuspended with FACS buffer (PBS containing 0.2 % BSA, Sigma-Aldrich) for flow cytometry analyses. For phospho-STAT staining, primary mouse T cells were rested in T cell medium lacking IL-2 for 24 h before signaling assays. Cells were plated in a 96-well round bottom plate in 50 μl T cell medium. Cells were stimulated with indicated cytokines for 20 min at 37 °C, and the reaction was terminated by fixation with 1.5% paraformaldehyde (PFA) for 15 min at room temperature with agitation. Cells were washed and permeabilized with ice-cold 100% methanol for 60 min on ice. Afterward, cells were washed with FACS buffer before staining with pSTAT antibodies for 1 h at 4 °C in the dark. Cells were washed and resuspended in FACS buffer for flow cytometry analyses. For intracellular cytokine staining, cells were first stimulated by a Cell Stimulation Cocktail (protein transport inhibitors included, Invitrogen/Thermo Fisher Scientific) at 37°C for 5 h. After stimulation, cells were first stained for surface markers and Zombie Violet Fixable Dye (BioLegend), then fixed and permeabilized with a Cytofix/Cytoperm^™^ Fixation/Permeabilization Solution Kit (BD Biosciences). Intracellular staining with indicated antibodies was performed following the manufacturer’s protocol. For transcription factor, Ki-67, or BrdU staining, cells were first stained for surface markers and Zombie Violet Fixable Dye. Next, cells were fixed and permeabilized with a Foxp3/Transcription Factor Staining Buffer Set (eBioscience) for transcription factor and Ki-67, or BD Pharmingen^™^ APC BrDU Kit (BD Biosciences) for BrdU staining according to the manufacturer’s instructions, followed by incubation with indicated antibodies for intracellular staining. For cytokine analysis, supernatants from tumor organoids or sorted mouse TILs stimulated with Cell Stimulation Cocktail (Invitrogen/Thermo Fisher Scientific) at 37°C for 5 h were collected and analyzed using the LEGENDplex^™^ Human CD8/NK Panel (BioLegend) or LEGENDplex^™^ Mouse Th Cytokine Panel (BioLegend) following the standard protocol provided with each kit. Cells or beads were detected using an CytoFlex (Beckman Coulter). Analyses were performed using FlowJo (v 10.10.0) or LEGENDplex Data Analysis Software Suite.

For experiments performed at the University of Pennsylvania, Cells were washed 1 X PBS and stained with amine-reactive dye Zombie NIR for 10 minutes to assess cell viability, followed by a chemokine receptor antibody cocktail in PBS with 2% FBS, 5 mM EDTA for 45 minutes at 37C. surface stain was performed by an antibody cocktail in SM for 45 min at room temperature. Fixation and permeabilization were performed using the Foxp3 Fixation/Permeabilization Concentrate and Diluent kit for 30 minutes at 4C. Permeabilization and intracellular staining with antibody cocktails were done overnight at 4C. Samples were run on Cytek Aurora spectral cytometer and SpectroFlo software. Data were analyzed with FloJo software.

For immunophenotyping of experiments investigating the IL-10/2-Trikine: C57BL/6 mice were inoculated s.c. with 5 × 10^5^ B16F10 tumor cells, with or without i.v. adoptive transfer of 5 × 10^6^ pmel T cells on day 9 post-tumor inoculation. Mice then received i.p. administration of mono-IL-10, IL-10/2-trikine (3 μg functional cytokine dose per dose), or PBS on days 9 and 11. On day 13, mice were sacrificed, and tumors and spleens were collected. For tetramer staining in the PD-1 combination study, C57BL/6 mice were inoculated s.c. with 5 × 10^5^ B16F10 tumor cells and received i.p. administration of mono-IL-10, IL-10/2-trikine (3 μg functional cytokine dose per dose), or PBS starting on day 9, every other day until day 15. Anti-PD-1 antibody (BioXcell, RMP1-14) was administered via i.p. injection on days 11 and 15 at a dose of 200 μg per dose. On day 16, mice were sacrificed, and tumors were collected. For BrdU and Ki-67 staining in the KP1 SCLC model, B6129SF1/J mice were inoculated s.c. with 5 × 10^5^ KP1 cells and received i.p. administration of mono-IL-10, IL-10/2-trikine (3 μg functional cytokine dose per dose), or PBS on days 7 and 9. Mice received i.p. injections of bromodeoxyuridine (BrdU; 2 mg/200 μL, BD Pharmingen^™^) on days 9 and 10. On day 11, mice were sacrificed and tumors were collected. For tumor-infiltrating lymphocyte (TIL) analysis in the PD-1 combination study in the KP1 SCLC model, B6129SF1/J mice were inoculated s.c. with 5 × 10^5^ KP1 cells and received i.p. administration of mono-IL-10, IL-10/2-trikine (3 μg functional cytokine dose per dose), or PBS every other day from day 7 to day 17. Mice also received i.p. injections of anti-PD-1 antibody (BioXcell, 200 μg per dose, RMP1-14) on days 9, 13, and 17. On day 18, mice were sacrificed and tumors were collected. Collected tumors were weighed, mechanically minced, and digested in RPMI-1640 medium supplemented with collagenase type IV (1 mg/ml, Gibco/Thermo Fisher Scientific), dispase II (100 μg ml^−1^, Sigma-Aldrich), hyalurondase (100 μg ml^−1^, Sigma-Aldrich), and DNase I (100 μg ml^−1^, Sigma-Aldrich) at 37 °C for 60 min. RBC lysis was performed on the digested tumor samples with ACK lysing buffer. Tumor infiltrating leukocytes were then enriched by Percoll (Cytiva) density gradient centrifugation, resuspended in PBS with BSA (0.2%, wt/v), stained with indicated antibodies, and analyzed by flow cytometry. Spleens were ground and filtered through a 70-μm strainer (Fisher Scientific). RBC lysis was performed on the spleen samples with ACK lysing buffer (2 ml per spleen, Gibco/Thermo Fisher Scientific) and then resuspended in PBS with BSA (0.2%, wt/v). TDLNs were ground and filtered through a 70-μm strainer (Fisher Scientific) and then resuspended in PBS with BSA (0.2%, wt/v). Cells collected from spleens and tumors were stained with the indicated antibodies and analyzed on a CytoFLEX flow cytometer (Beckman Coulter).

### Chronic stimulation via BiTE experiments

Naïve CD8+ T cells were isolated using the EasySep^™^ Human Naïve CD8+ T Cell Isolation Kit II from PBMCs from healthy donors. These cells were co-cultured with NALM6-CBG^+^-GFP^+^ target cells at a 1:1 effector-to-target (E:T) ratio in the presence of 5 ng/mL of anti-hCD19-CD3 bispecific antibody in RPMI-1640 media supplemented with 10% FBS, 1x non-essential amino acids, 1x Sodium Pyruvate, 10mM Hepes, 1x GlutaMAX, 100U/mL penicillin/streptomycin (cRPMI). To maintain a consistent E:T ratio throughout the 25-day assay, NALM6 cells and the bispecific antibody were replenished every 48 hours. Additionally, cytokine concentrations were adjusted every 48 hours to maintain appropriate levels within the culture.

### Repeated antigen challenge experiments

NY-ESO-1-targeted T cells were generated via retroviral transduction as previously described (8). Briefly, CD8+ T cells were isolated from human PBMCs via MACS (Miltenyi). T cells were activated with CD3/CD28 Dynabeads (Gibco) for 48 hours, followed by transduction via retrovirus containing a pMSGV1 vector containing the 1G4 TCR (64). Five days after transduction, Dynabeads were removed and cells continued to expand for a total of 10 days.

NY-ESO-1-TCR T cells were then seeded at a 1:1 ratio with M407 target cells in the presence of Trikine, cytokine, or no cytokine control, replenished with fresh cytokine and M407 cells every 48 hours. For real-time killing analysis, cells were taken from this continuous culture and seeded at a 1:1 ratio with nRFP+ M407 cells for monitoring and analysis via IncuCyte Live Cell Analysis (Essen Bioscience) as previously described (8). For phenotypic analysis, cells were taken from continuous culture and underwent immunophenotyping by FACS as described.

### Mouse IL-21 DN development

We previously developed human IL-21 variants that attenuated their pSTAT3 activation strength via mutations at the binding interface with γ_c_, specifically amino acid Q116 (23). We modeled mouse IL-21 in complex with mouse extracellular domains of IL-21Ra and γ_c_ using AlphaFold and then aligned the model with the human IL-21 signaling complex structure (PDB 8ENT) using ChimeraX. From the alignment, we predicted the structural analog to Q116 of human IL-21 to be Q121 of mouse IL-21. Mouse IL-21 with the mutations Q121L, Q121D, Q121T, and Q121I were produced and tested in YT-1 cells for pSTAT3 signal as described above.

### Mice

For experiments performed at Stanford University, five- to six-week-old Thy1.2^+^ C57BL/6 (C57BL/6J) mice and B6129SF1/J (F1, Strain #101043) mice were purchased from Jackson Laboratory. TCR-transgenic Thy1.1^+^ pmel-1 (pmel) mice (B6.Cg-Thy1a/Cy Tg(TcraTcrb)8Rest/J) were originally purchased from the Jackson Laboratory and maintained in the Stanford University-Lorry Lokey (SIM1) Facility. Mice were housed in animal facilities approved by the Association for the Assessment and Accreditation of Laboratory Care. Experimental procedures in mouse studies were approved by the Institutional Animal Care and Use Committee (IACUC) at the Stanford University (animal protocol ID 32279 and 13565) and at the Dana-Farber Cancer Institute (animal protocol ID 14019) and performed in accordance with the guidelines.

For experiments performed at University of California, Berkeley, Mice were maintained at the University of California, Berkeley. C57BL/6J mice were purchased from the Jackson Laboratory. All mice used were between 8 and 20 weeks of age. All experiments were approved by the University of California (UC) Berkeley Animal Care and Use Committee and were performed in adherence to the NIH Guide for the Care and Use of Laboratory Animals (National Research Council Committee for the Update of the Guide for the and Use of Laboratory, 2011).

### Bulk RNA-seq experiment

Mouse T cells were pre-activated with IL-2 as described and rested in the absence of serum or cytokine overnight. Cells were then incubated with 100 nM IL-2, IL-21, or Trikine for 24 hours. For each condition, we performed 3 technical replicates. Total RNA was extracted from 1-2.5 million cells per condition using a RNeasy Micro Kit (Qiagen) followed by mRNA library preparation (poly-A enrichment) according to manufacturer instructions. Each library was sequenced on the NovaSeq X Plus PE150 resulting in approximately 12 x 10^6^ reads per sample. FastQC was used to check the quality of fastq files. Sequences were mapped to the GRCm38/mm10 reference genome with RSubread, and gene features were quantified with featureCounts. Differential expression (DE) analysis was conducted with the DESeq2 package in R. Geneset enrichment analysis (GSEA) was conducted with the fgsea package in R. ENCODE ChIP-seq genesets were retrieved from the ChEA3 web portal (https://maayanlab.cloud/chea3/index.html#content4-13). Human genes were converted into mouse gene homologues based on the homologene database (ftp://ftp.ncbi.nih.gov/pub/HomoloGene/build68/). ChIP-seq analysis on STAT3-dependent genes were retrieved from a previous publication and processed (18). Heatmap was created using the ComplexHeatmap package in R, using Z-transformed VST-normalized read counts for each gene.

### *In vivo* toxicity experiments

To assess toxicity observed in healthy mice, female mice were treated (i.p.) with 10 μg of functional cytokine every other day for 4 treatments. Mouse weight was measured at the time of each dose administration. 24 hours after the 4^th^ dose, a 30 second video was taken of each mouse, and motility tracking was conducted and quantified as previously described (5). A necropsy was then performed, at which time spleens, livers, lungs, pancreases, and blood were collected. Spleens were weighed and blood samples were subsequently centrifuged to separate blood cells from serum. Serum was collected and stored at −80C until time of analysis. The livers, lungs, and pancreases were immediately stored in a 10% neutral buffered formalin solution (Millipore Sigma, HT501128). Fixed livers, lungs, and pancreases were processed, embedded, blocked, cut, and stained for Hematoxylin and Eosin (H&E) by Histo-Tec Laboratory Inc., and stained slides were imaged on a Leica DM2000 histology scope at the Stanford Cell Sciences Imaging Facility. Inflammation scoring for histological samples was performed in a blinded fashion from 0 (none) to 4 (severe) lymphocyte infiltration based on previously published methodology (65).

Indicated cytokines were quantified using the ELISA MAX Standard Set Mouse IFNγ kit (Biolegend) or LEGENDplex Mouse Inflammation Panel (Biolegend) according to manufacturer instructions. Serum Amyloid A was quantified using the Mouse SAA ELISA Kit (Invitrogen) according to manufacturer instructions. ELISA assay plates were read on a SpectraMax Paradigm plate reader using SoftMax Pro v7.1. LEGENDplex samples analyzed by flow cytometry using a Beckman-Coulter Cytoflex S and FlowJo software (v10.10.0). Livers, lungs, and pancreases we

For cell depletion toxicity experiments, male and female mice received 400 μg doses (i.p.) of cell-type-specific depletion antibodies at 24 hours prior to the first and the third cytokine doses. The following antibodies were purchased from BioXCell for depletion studies: anti-CD8α (2.43, BE0061), anti-CD4 (GK1.5, BE0003-1), and anti-NK1.1 (PK136, BE0036).

### Antibodies and reagents for flow cytometry

The following antibodies or staining reagents were purchased from BioLegend: mouse CD16/32 (93, 101302), mouse CD90.1/Thy-1.1 (OX-7, 202533), mouse CD8β (YTS156.7.7, 126622), mouse SCA-1 (D7,108142), mouse CD62L (MEL-14, 104428), mouse CD44 (IM7, 103030), mouse PD-1 (29F.1A12, 135225), mouse/human Granzyme B (QA16A02, 396438), human/mouse Granzyme B (QA16A02, 372213), mouse IFN-γ (XMG1.2, 505850), mouse TCF1 (7F11A10, 655204), mouse TIM-3 (RMT3-23, 119721), mouse TIM-3 (RMT3-23, 119721), mouse CD45.2 (104, 109839), mouse CD3 (17A2, 100204), mouse CD4 (RM4-5, 100512), mouse NK1.1 (PK136, 108736), mouse CD11b (M1/70, 101228), mouse F4/80 (BM8, 123107), mouse CD11c (N418, 117348), mouse I-A/I-E (M5/114.15.2, 107622), mouse CD103 (2E7, 121405), mouse Ly-6C (HK1.4, 128049), mouse Ly-6G (1A8, 127649), mouse CD19 (6D5, 115519), mouse CD45.2 (104, 109807), mouse CD4 (GK1.5, 100434), mouse IL-10R (1B1.3a, 112705), mouse IL-2Rβ (5H4, 105911), mouse/human ICOS (C398.4A, 313538), mouse TNF-α (MP6-XT22, 506322), mouse Ki-67 (16A8, 652413), mouse CXCR6 (SA051D1, 151116), mouse Bcl2 (BCL/10C4, 633506), mouse Bcl6 (7D1, 358512), mouse IFN-γ (XMG1.2, 505832), mouse/rat/human CD27 (LG.3A10, 124215), mouse PD-1 (RMP1-30, 109119), mouse Perforin (S16009A, 154309), mouse CD45 (2D1, 103128), mouse Ki-67 (16A8, 652421), mouse CD45 (2D1, 100236), mouse Perforin (S16009A, 154309), mouse CD27 (LG.3A10, 123215), mouse CD3 (UCHT1, 108139), mouse CD27 (LG.3A10, 124225), mouse CD45 (2D1, 100235), mouse CD4 (RM4-5, 100509), mouse CD8b (YTS156.7.7, 126622), mouse CD45 (30-F11, 103128), mouse CD44 (IM7, 103027), mouse CD25 (PC61, 102029), mouse CD62L (MEL-14, 104453), mouse CD223 (LAG-3) (C9B7W, 125227), mouse IL-2 (JES6-5H4, 503821), mouse IFN-γ (XMG1.2, 505849), mouse Granzyme A (3G8.5, 149703), mouse Perforin (S16009A, 154309), mouse CD69 (H1.2F3, 104543), mouse CD4 (RM4-5, 100509), mouse TNF-α (MP6-XT22, 506307), mouse CD69 (H1.2F3, 104539), mouse CD4 (RM4-5, 100536), mouse CD8a (53-6.7, 100725), mouse CD3 (17A2, 100232), mouse CD25 (3C7, 101907), mouse CD62L (MEL-14, 104407), mouse CD69 (H1.2F3, 108749), mouse CD95 (Fas) (SA367H8, 152603), mouse CD127 (IL-7Rα) (A7R34, 135041), mouse Ly-6A/E (Sca-1) (D7, 108114), mouse TNF-α (MP6-XT22, 506307), mouse IL-2 (JES6-5H4, 503807), mouse CD8a (53-6.7, 100741), mouse CD4 (RM4-5, 100543), human CD3 (UCHT1, 300408), human CD8 (SK1, 344712), human CD4 (OKT4, 317408), human TNF-α (Mab11, 502928), human IFNγ (4S.B3, 502530), human CD122 (IL-2Rβ) (TU27, 339011), human CD25 (IL-2Rα) (BC96, 302613), human CD69 (FN50, 310931), human CD279 (PD-1) (A17188B, 621613), human CD223 (LAG-3) (C9B7W, 369321), human CD132 (TUGh4, 338606), human CD3 (OKT3, 317326), human CD45RA (HI100, 304150), human CD62L (DREG-56, 304829), human CD39 (A1, 328235), human CD8a (HIT8a, 300928), human CD4 (RPA-T4, 300506), human CD45RO (UCHL1, 304227), human CD8 (SK1, 344739), human CD122 (IL-2Rβ) (TU27, 339010), human CD62L (DREG-56, 304829), human CD27 (O323, 302838), human CD62L (DREG-56, 304803), human CD8a (SK1, 344739), human CD62L (DREG-56, 304829), human CD4 (RPA-T4, 300506), Mouse TruStain FcX^™^ (101319), Human TruStain FcX^™^ (422302) and Zombie Violet^™^ Fixable Viability Kit (423114). The following antibodies or staining reagents were purchased from BD Biosciences: pSTAT3 (4/P-STAT3, 557815), pSTAT5 (47/Stat5, 612599), mouse CD8a (53-6.7, 563898), mouse CD44 (IM7, 563058), mouse CD45.2 (104, 561875), human IFN-γ (4S.B3, 554551), human CD45RA (HI100, 740298), human Granzyme B (GB11, 563389), human CD4 (SK3, 566355), human PD-1 (EH12.1, 566460), human Ki-67 (B56, 561277), and BD Pharmingen^™^ APC BrdU Kit (552598). The following antibodies were purchased from Cell Signaling Technology: human/mouse phospho-STAT1 (Tyr701) (58D6, 8009), human/mouse TCF1/TCF7 (C63D9, 6444), human/mouse TCF1/TCF7 (C63D9, 6709S). The following antibodies were purchased from Invitrogen: mouse NK1.1 (PK136, 12-5941-82), mouse Foxp3 (FJK-16s, 12-5773-82), mouse/human Ki-67 (SolA15, 11-5698-80), mouse/human CD27 (LG7F9, 12-0271-82), mouse/human Ki-67 (SolA15, 46-5698-82), mouse perforin (eBioOMAK-D, 12-9392-82), human/mouse Foxp3 (PCH101, 35-4776-42), and mouse CD3e (145-2C11, 45-0031-82). The following antibodies were purchased from Miltenyi: mouse/human TOX (REA473, 130-118-335) and mouse/human TOX (REA473, 130-120-716). H-2D^b^ gp100 tetramer (EGSRNQDWL-PE) was obtained from the NIH Tetramer Core Facility. DAPI was purchased from Thermo Fisher.

### Mouse NK cell *ex vivo* assays

For cytotoxicity assays, the NK sensitive target cells were MC38 tumor cells that were MHC I deficient due to targeted disruption of the B2m gene (66). Cytotoxicity by splenocytes was assessed with a standard 4-hour ^51^Cr-release assay as described (66). For stimulation assays to assess NK degranulation and cytokine production, single cell suspensions of splenocytes were incubated for 4 hours in wells coated with antibodies for the NKp46 activating receptor, or isotype control antibodies, in DMEM medium containing Alexa Fluor-conjugated CD107a antibodies, 2.4G2 hybridoma supernatant to block FcγRII/III receptors, brefeldin A (Biolegend), and monensin (Biolegend) before performing surface and intracellular staining followed by flow cytometric analysis, gating on CD3-NK1.1+ cells.

### *In vivo* anti-tumor therapy studies with IL-2/21-Trikine treatment

Thy1.1+ pmel cells were isolated from the spleens of pmel mice and cultured with 1 μg/ml gp100 and 10 nM cytokine for 2 days. After 2 days, the media was replaced daily with 10 nM cytokines until day 6. On day 6 of preconditioning, live cell enrichment was performed with Ficoll. Cells were prepared in PBS for ACT and leftover cells were used for analysis by flow cytometry.

On the day of pmel cell harvest, 3x10^5^ B16F10 cells were subcutaneously injected into the right flank of C57BL/6 mice. 5 days after tumor injection, mice were lymphodepleted using 5 or 6 Gy radiation (for the high and low dose regimens, respectively) and received 1x10^7^ preconditioned pmel cells via tail vein injection. On the day of ACT and for three additional doses spaced every 48 hours, mice receiving IL-2 or IL-2 + IL-21 preconditioned cells received an i.p. injection of IL-2. Mice receiving Trikine preconditioned cells received i.p. injections of Trikine with the same dosing schedule. The doses of i.p. injected protein were 5 μg /dose for the high dose regimen and 2.5 μg /dose for the low dose regimen.

Tumor size and weight change were measured every other day. For tumor growth and survival studies, mice were euthanized on the basis of poor body condition and/or >20% weight loss. For analysis of tissues, mice were sacrificed on day 18 following tumor injection for dLN and tumor harvest.

### *In vivo* anti-tumor therapy studies with IL-10/2-Trikine treatment

C57BL/6 mice bearing established subcutaneous B16F10 tumors received intraperitoneal (i.p.) administration of mono-IL-10, IL-10/2-Trikine (3 μg functional cytokine dose per dose), or PBS control every other day from day 5 to day 19. For combination treatment, anti-PD-1 antibody (BioXcell, RMP1-14) was administered via i.p. injection on days 7, 11, 15, and 19 at a dose of 200 μg per dose.

For adoptive cell therapy, C57BL/6 mice bearing established subcutaneous B16F10 tumors received an intravenous (i.v.) adoptive transfer of 5 × 10^6^ pmel T cells on day 5, followed by i.p. administration of IL-2, mono-IL-10, IL-10/2-Trikine (3 μg functional cytokine dose per dose), or PBS control every other day until day 19.

For the KP1 SCLC tumor model, B6129SF1/J mice received i.p. administration of mono-IL-10, IL-10/2-Trikine (3 μg functional cytokine dose per dose), or were left untreated every other day from day 5 to day 19. Anti–PD-1 antibody was administered via i.p. injection on days 7, 11, 15, and 19 at a dose of 200 μg per dose. For immune cell depletion studies, B6129SF1/J mice bearing established KP1 tumors were treated on day 7 post-inoculation with intraperitoneal (i.p.) administration of IL-10-trikine (3 μg functional cytokine dose) and anti-PD-1 antibody as described above. To deplete specific immune cell subsets, mice received i.p. injections of the following antibodies one day prior to treatment: anti-CD8 (YTS 169.4, BioXcell, 400 μg per mouse), anti-CD4 (YTS 177, BioXcell, 400 μg per mouse), a combination of anti-CD8 and anti-CD4 (400 μg each), or anti-NK1.1 (PK136, BioXcell, 400 μg per mouse). Two additional doses of the respective antibodies were administered during treatment to maintain depletion.

For the 6694C2 PDAC model, male and female C57BL/6 mice (6 to 8 weeks old) were subcutaneously inoculated with 250,000 6694C2 cells in the right flank. Four days post-inoculation, mice were administered treatments according to the following dosing regimens: one group received intraperitoneal injections mono-IL10 (26 μg/mouse, n=10) every two days; a second group received IL-10 Trikine (30.6 μg/mouse, n=10) on the same schedule. A third group received a combination of anti-PD1 and anti-CTLA-4 antibodies (Thermo Fisher Scientific Cat# 50562455; Bioxcell Cat# BE0146; 150 μg each per mouse, n=10) intraperitoneally every three days. All treatments were continued until tumors reached the humane endpoint volume of 1000 mm^3^. All mice were monitored for changes in body weight.

Post-therapy survival of mice was monitored for at least two-month post-tumor inoculation. Mice were euthanized when body weight loss was beyond 15% of baseline weight, tumor area reached 200 mm^2^, or any signs of discomfort were detected by the investigators or as recommended by the caretaker who monitored the mice every day.

### Amylase Assay

Blood serum was collected from both untreated mice and mice treated for five days. Amylase activity was measured using the Pointe Scientific Liquid Amylase (CNPG3) Reagent Set (Catalog #A7564120). Following the manufacturer's instructions, reagents were first equilibrated to room temperature. In a 96-well plate, 100 μL of the amylase reagent was aliquoted per well and pre-incubated at 37 °C for 5 minutes. Subsequently, 2.5 μL of each serum sample was added to the wells. The absorbance at 405 nm was measured spectrophotometrically every minute for three minutes. Enzyme activity (U/L) was calculated using the following formula: Amylase Activity = (ΔA_405_/min × Total Reaction Volume (μL) × 1000) / (12.9 × Sample Volume (μL) × Light Path (1 cm)), where 12.9 is the millimolar absorptivity of 2-chloro-p-nitrophenol and ΔA_405_/min is the mean change in absorbance per minute.

### Aspartate Aminotransferase (AST) Assay

Blood samples from untreated and treated mice were centrifuged at 5000 rcf for 5 minutes at room temperature to separate the serum. The supernatant was collected and diluted 1:250 in the provided sample diluent NS (Mouse AST SimpleStep ELISA^®^ Kit, Abcam, cat# ab263882). Standards were prepared according to the manufacturer's protocol. Subsequently, 50 μL of each diluted sample or standard was added in duplicate to the appropriate wells of a 96-well plate. Then, 50 μL of the antibody cocktail was added to each well. The plate was sealed and incubated for 1 hour at room temperature on a plate shaker set to 400 rpm.

Following incubation, the plate was washed three times with 1X Wash Buffer PT. Next, 100 μL of TMB Development Solution was added to each well, and the plate was incubated in the dark for 10 minutes. The enzymatic reaction was stopped by adding 100 μL of Stop Solution to each well. The absorbance of each well was immediately measured at 450 nm using a microplate reader.

For data analysis, a standard curve was generated by plotting the mean absorbance (y-axis) against the known concentration of each standard (x-axis). The concentration of aspartate aminotransferase (AST) in each unknown sample was interpolated from this curve and multiplied by the sample dilution factor.

### Alanine Aminotransferase (ALT) Assay

Blood samples from untreated and treated mice were centrifuged at 5000 rcf for 5 minutes at room temperature to separate the serum. The supernatant was collected and diluted 1:250 in the provided 1X Cell Extraction Buffer PTR (Mouse ALT SimpleStep ELISA^®^ Kit, Abcam, cat# ab282882). Standards were prepared according to the manufacturer's protocol. Subsequently, 50 μL of each diluted sample or standard was added in duplicate to the appropriate wells of a 96-well plate. Then, 50 μL of the antibody cocktail was added to each well. The plate was sealed and incubated for 1 hour at room temperature on a plate shaker set to 400 rpm.

Following incubation, the plate was washed three times with 1X Wash Buffer PT. Next, 100 μL of TMB Development Solution was added to each well, and the plate was incubated in the dark for 10 minutes. The enzymatic reaction was stopped by adding 100 μL of Stop Solution to each well. The absorbance of each well was immediately measured at 450 nm using a microplate reader.

For data analysis, a standard curve was generated by plotting the mean absorbance (y-axis) against the known concentration of each standard (x-axis). The concentration of alanine aminotransferase (ALT) in each unknown sample was interpolated from this curve and multiplied by the sample dilution factor.

### Creatinine Assay

Serum was collected from untreated mice and from mice following a 5-day treatment period. All reagents from the Creatinine Assay Kit (Colorimetric/Fluorometric, Abcam, cat# ab65340) were equilibrated to room temperature. A 1 nmol/μL creatinine standard was prepared by diluting 10 μL of the supplied 100 mM creatinine standard in 990 μL of assay buffer. Subsequently, 50 μL of the prepared standards were aliquoted into a 96-well plate. Serum samples were diluted 1:10 in assay buffer, and 50 μL of each diluted sample was added to the appropriate wells.

A master reaction mix was prepared according to the manufacturer's protocol, containing assay buffer, creatinase, sarcosine enzyme mix, creatininase, and the OxiRed probe. Then, 50 μL of the reaction mix was added to each well containing standard or sample. The plate was mixed thoroughly and incubated at 37 °C for 1 hour. Following incubation, the absorbance was measured at 570 nm using a microplate reader.

For data analysis, a standard curve was generated by plotting the absorbance of the known standards. The creatinine concentration in each sample was determined by interpolating the sample absorbance value from the standard curve to calculate the amount of creatinine (in nmol) in the well. This value was then divided by the sample volume and multiplied by the initial sample dilution factor to obtain the concentration in nmol/ml. Final concentrations were converted to mg/dL by multiplying the nmol/mL value by a factor of 0.0113, based on the molecular weight of creatinine (113.12 g/mol).

### Cell sorting

B6129SF1/J mice were inoculated s.c. with 5 × 10^5^ KP1 cells and received i.p. administration of IL-10/2-Trikine (3 μg functional cytokine dose per dose) every other day from day 7 to day 17, along with anti-PD-1 antibody (BioXcell, 200 μg/injection, RMP1-14) on days 7, 11, and 15. On day 18, mice were sacrificed and tumors were collected. Tumor-infiltrating leukocytes were enriched by density gradient centrifugation using Ficoll-Paque PLUS (Cytiva) and then stained with surface markers and DAPI (Thermo Fisher Scientific). CD25^−^PD-1^+^ICOS^+^ CD4^+^ T cells, CD25^−^ non-DP CD4^+^ T cells, CD25^+^IL-7Rα^−^ Tregs, and CD8^+^ T cells were sorted were sorted using an Aria II sorter (BD Biosciences) at the Stanford Share FACS Facility for cytokine profiling or used for adoptive cell transfer.

### Single cell RNA-sequencing sample preparation

For the low dose regimen of sIL-2, sIL-2 + IL-21, or IL-2/21-treatment in the B16F10 + pmel ACT model, tumors from mice were harvested on day 18 following tumor injection. Tumors were minced and dissociated using a gentleMACS Octo Dissociator. TILs were collected for sequencing by sorting for live, single cell, Thy1.1+CD8+ cells on a Sony Cell Sorter SH800S.

For the KP mouse tumor model, B6129SF1/J mice were inoculated s.c. with 5 × 10^5^ KP1 cells and received i.p. administration of mono-IL-10 or IL-10/2-Trikine (3 μg functional cytokine per dose) every other day from day 7 to day 19, along with anti-PD-1 antibody (BioXcell, 200 μg per dose, clone RMP1-14) on days 7, 11, 15, and 19. On day 19, mice were sacrificed and tumors were collected. Tumor-infiltrating leukocytes were enriched by density gradient centrifugation using Ficoll-Paque PLUS (Cytiva), followed by surface marker and DAPI staining (Thermo Fisher Scientific). Live CD45^+^ leukocytes were sorted using a Sony SH800 Cell Sorter (Sony Biotechnology).

For both B16F10 and KP tumor model experiments, sorted cells underwent single-cell encapsulation using the Chromium Single Cell System and associated reagents. A Chromium Next GEM Chip G was loaded with the appropriate cell concentration, and sequencing libraries were prepared following the manufacturer’s protocol with 10x Genomics reagents. In brief, an emulsion was formed to encapsulate individual cells within droplets, along with reagents and gel beads containing unique molecular identifiers (UMIs), reverse transcription components, and cell barcoding oligonucleotides. After droplet disruption, cDNA synthesis and amplification were performed. For the 3′ Gene Expression library, the amplified cDNA was fragmented, ligated to sequencing adapters, and enriched via PCR. The resulting 3′ Gene Expression libraries were sequenced on a NovaSeq 6000, achieving a depth of over 20,000 paired-end reads per cell. The 10x Genomics Cloud was used to generate a gene expression matrix; sequenced reads were uploaded to the Cell Ranger Count v9.0.0 pipeline and processing using the Mouse (GRCm39) 2024-A genome as reference and using following settings:

checkLibraryCompatility: true; chemistry: auto; createBam: false; disableAbAggregateDetection: false; includeIntrons: true; noSecondaryAnalysis:false; skipCellAnnotation:false.

For the PDAC tumor model, mice were inoculated s.c. with 2.5 × 10^5^ 6694c2vTRP1 cells and received i.p. injections of PBS, mono-IL-10 (26 μg/mouse, n = 5), or IL-10/2-Trikine (30.6 μg/mouse, n = 5) every other day starting on day 8, for a total of two doses. On day 14, mice were sacrificed and tumors were collected. Tumors were minced and dissociated at 37 °C in digestion buffer [RPMI-1640, 2% FBS, 0.2 mg/mL Collagenase P (Roche), 0.2 mg/mL Dispase (Gibco), and 0.1 mg/mL DNase I (Roche)], with repeated pipetting; supernatants containing liberated cells were collected every 10 minutes and quenched on ice in 50 mL conical tubes containing cold FACS buffer (PBS with 2% FBS and 0.5 mM EDTA). Tumor suspensions were passed through a 70-μm cell strainer, washed with PBS, and centrifuged. The resulting cell pellet, containing tumor debris and infiltrating immune cells, was resuspended in isolation buffer (RPMI-1640, 1% FBS) and subjected to CD45^+^ bead isolation (STEMCELL #18945) according to the manufacturer’s instructions. Isolated CD45^+^ TILs from each group were stained with TotalSeq anti-mouse hashtag antibodies 1–5 (BioLegend), pooled into a single sample, and processed for single-cell encapsulation using 10x Genomics Chromium GEM-X Single Cell 5’ Reagent Kits. Approximately 35,000 cells in total were loaded per sample, and gene expression and hashtag libraries were generated according to the manufacturer’s protocol. The resulting 5’ gene expression and hashtag libraries were sequenced on an Illumina NovaSeq X Plus (single-cell configuration) operated by Azenta/Genewiz, achieving >25,000 (gene expression) or >5,000 (hashtag) paired-end reads per cell. The Cell Ranger “count” pipeline (10x Genomics, v9) was used with default parameters to align reads to the mouse GRCm39 reference genome and generate single-cell feature count matrices for each library.

### Analysis of single cell RNA-sequencing data

For the low dose regimen of sIL-2, sIL-2 + IL-21, or IL-2/21-treatment in the B16F10 + pmel ACT model, the gene expression matrix was processed and analyzed using Seurat (version 5.1.0). For quality control, we excluded cells that contained less than 500 or more than 120,000 reads for genes. We also excluded cells in which more than 20% of transcripts were derived from mitochondrial RNA. These preprocessing left 30,856 cells in total (7,760, 13,299, and 9,797 cells for IL-2, Trikine, and IL-2 + IL-21 treatment groups, respectively). Unsupervised clustering analysis were performed using Monocle 3 (version 1.3.7). Clusters were manually annotated using the distribution of key T cell differentiation markers among the top 250 differentially expressed genes across clusters. Differential expression (DE) analysis between clusters and between treatment groups was performed via the Wilcoxon’s rank sum test implemented in Seurat’s FindMarkers function.

For the KP mouse tumor model, the gene expression matrix was first processed using Seurat (version 5.1.0). For each dataset, we excluded cells containing fewer than 200 genes (to remove debris, empty droplets, and low-quality cells) as well as cells in which >20% of transcripts were derived from mitochondrial RNA. These preprocessing steps left 58566 cells in total (18678, 17765, and 22123 cells for the mono-IL-10, IL-10-trikine, and IL-10/2-Trikine + anti-PD-1 treatment groups, respectively). For immune subset annotation, we first used the SingelR pipeline with the MouseRNAseqData from the celldex package as a reference. We then performed unsupervised clustering using Seurat, and annotated each cluster based on the aggregated SingleR prediction scores and manual inspection of canonical marker genes. Unidentifiable cells were excluded from subsequent analyses, leaving 54788 cells in total (17608, 16685, and 20495 cells, respectively). T cells (6191, 6915, and 8829 cells, respectively; 21935 cells in total) were further analyzed using Monocle 3 (version 1.3.7). S and G2M phase scores were computed with Seurat’s cell cycle gene lists converted from human to mouse orthologs. The cell cycle delta scores were computed by subtracting G2M scores from S scores to better separate cycling and non-cycling cells. Unsupervised clustering was performed with Monocle 3’s standard workflow, with regression to the percentage of mitochondrial transcripts and the cell cycle delta scores. Differential expression (DE) analysis between clusters and between treatment groups was performed using the Wilcoxon rank-sum test implemented in Seurat’s FindMarkers function. For heatmap visualization of cluster marker genes, representative genes for each cluster were manually selected from the top 10 genes with the highest average log_2_ fold change. Gene set enrichment analysis (GSEA) was performed using the log_2_ fold-change ranking of DE genes between treatment groups (all clusters combined) via the fgsea package. Mouse Hallmark gene sets were retrieved from MSigDB.

For the PDAC tumor model, the filtered barcode matrix was processed and analyzed using Seurat (v5.1.0). For quality control, we excluded cells with fewer than 1,000 detected genes and those with >10% mitochondrial reads. Counts from each sample were log-normalized, scaled, and subjected to dimensionality reduction by Principal Component Analysis (PCA). To minimize batch effects across mice, we used Harmony (v1.2.1) for integration, and generated Uniform Manifold Approximation and Projection (UMAP) embeddings from the top 50 Harmony dimensions. For T cell sub-clustering, variable features were re-identified for each sample, excluding TCR and Ig variable region genes, and clusters were manually annotated. Core STAT5 targets list (*Gzmb, Prf1, Nkg7, Gzma, Klrd1, Klrb1c, Klra7, Ccr5, Ly6a, Thy1*) was found from the literature (67-70). Differential expression (DE) analysis between clusters and between treatment groups was performed by summing gene counts across all cells and using the DESeq2 (v1.46.0) package. Gene set enrichment analysis (GSEA) was conducted using log_2_ fold-change–ranked DE genes between treatment groups (all clusters combined) with the fgsea (1.32.4) package. Mouse Hallmark gene sets were obtained from MSigDB.

### Fresh human melanoma specimens

Human melanoma tissue specimens were obtained from patients undergoing treatment, in accordance with an approved tissue collection protocol (IRB #65607) sanctioned by the Stanford University Institutional Review Board. Written informed consent was obtained from all participants. Samples were collected from adult individuals of both genders, and no differences in organoid formation capacity were observed between male and female samples. All surgical specimens were histologically confirmed as melanoma prior to processing.

### Human melanoma tumor sample processing and organoid culture

Melanoma tissue was collected in RPMI medium (Gibco) supplemented with 10% FBS (Gibco) and transported on ice for processing within 24 hours for organoid generation, following previously established protocol with minor modifications (48). For the TIL expansion experiment, tissues were minced with sterile scissors and dissociated using a gentleMACS Octo Dissociator. Following dissociation, live cell enrichment was performed with Ficoll. Cells were then plated at approximately 1 x 10^6^ cells/ml in T cell media supplemented with 10 or 28 nM of sIL-2, sIL-2 + IL-21, or IL-2/21-Trikine. Fresh cytokine was added every 2 days and full media changes were performed every 2-4 days. Cells were passaged every 2 to 4 days as wells reached confluency. Cells were analyzed by flow cytometry after 14 or 21 days in culture.

For tissues used in organoid culture, tissues were washed 2-3 times with DPBS (Gibco), and excess connective and vascular tissue was removed. The tissue was then finely minced on ice using a sterile scalpel, and the cell suspension was collected into a 15 mL tube and centrifuged at 1200 rpm for 5 minutes at 4 °C. The supernatant was discarded, and the cell pellet was resuspended in Matrigel (Corning). This was mixed with complete organoid culture medium at a 1:2 ratio and plated as 20 μL droplets in a 12-well plate. The Matrigel droplets were allowed to solidify at 37 °C for 15-20 minutes before adding 1 mL of organoid culture medium. The organoid culture medium consisted of 40% Advanced DMEM/F12 (Gibco), 50% Wnt3a, RSPO1, and Noggin conditioned media (L-WRN, ATCC), and 10% heat-inactivated FBS (Gibco). The medium was further supplemented with 1 mM HEPES (Invitrogen), 1x GlutaMAX (Invitrogen), 10 mM Nicotinamide (Sigma), 1 mM N-Acetylcysteine (Sigma), 1x B27 supplement without vitamin A (Invitrogen), 0.5 μM A83-01 (Tocris), 1x Penicillin-Streptomycin-Glutamine (Invitrogen), 10 nM Gastrin (Sigma), 50 ng/mL EGF (Peprotech), and 50 ng/mL FGF-1 (Peprotech). During the first two weeks of culture, 1x Normocin (InvivoGen) was added to prevent microbial contamination. Organoids were passaged approximately once a week by incubation in TrypLE Express (Gibco) for 5-10 minutes at 37 °C to dissociate them into single cells, followed by replating in fresh Matrigel. For long-term storage, organoids were cryopreserved either in 10% DMSO with 90% heat-inactivated FBS or in Recovery^™^ Cell Culture Freezing Medium (ThermoFisher) and maintained as biobanks.

### Organoid viability assay

For a proof-of-concept screening assay, selected compounds were tested for their effects on organoid viability. These included Nivolumab (Anti-PD-1, 6 μM), mono-IL-10 (30 nM), IL-10/2-Trikine (30 nM), and an untreated control. Single cells were seeded into 96-well plates coated with Matrigel at a density of 5x10^3^ cells per well and cultured for seven days to allow organoid formation. The compounds were diluted in culture medium and applied to the organoids across a range of concentrations, followed by a seven-day incubation. Cell viability was assessed using the CellTiter-Glo assay (Promega, G9681, Madison, WI, USA) according to the manufacturer’s protocol. Luminescence was measured on a Biotek plate reader using Gen4 software to determine whether the compounds enhanced cytotoxicity. Growth inhibition was calculated as a percentage relative to the untreated control. All assays were performed in triplicate.

### Cell killing assay

Tumor cells from patient T133 were isolated by passage and seeded into 12-well plates overnight. Autologous PBMCs from patient T133, expanded in vitro, were cocultured with T133 tumor cells at a 2:1 effector-to-target (E:T) ratio, with or without Nivolumab (anti-PD-1, 20 μg ml^−1^; Selleckchem), and in the presence or absence of mono-IL-10 or IL-10/2-Trikine (30 nM). After 48 hours of coculture, cells were harvested and analyzed by flow cytometry to assess tumor cell viability and T cell phenotype.

### Statistical analysis

Statistical analysis was performed using GraphPad Prism 10 (GraphPad software), except scRNA-seq data, which were analyzed with R (described above). All values and error bars are shown as mean ± SEM or ± SD as noted. Comparisons of two groups were performed by using two-tailed unpaired Student’s t test. Comparisons of multiple groups were performed by using one-way analysis of variance (ANOVA) with Tukey’s multiple-comparisons test unless otherwise indicated. Experiments that involved repeated measures over a time course, such as tumor growth were performed by using two-way ANOVA with Tukey’s multiple-comparisons post-test. Survival data were analyzed using the Log-rank (Mantel-Cox) test. No statistically significant (ns) differences were considered when *p*-values were larger than 0.05.

## Supplementary Material

mdar_v1

Supplemental

References #62-#70

Figs. S1 to S13

Reproducibility Checklist
