# Supplementary material for: Rewiring STAT signaling from the cell surface with Trikine immunotherapeutics
Source: Science. Author manuscript; Available in PMC 2026 Mar 7. (PMC12963926; doi:10.1126/science.adx9954)
Supplement: Supplemental [file NIHMS2144531-supplement-Supplemental.pdf]

Science
AAAS

Supplementary Materials for

Rewiring JAK/STAT signaling from the cell surface with Trikine immunotherapeutics

Authors: Grayson E. Rodriguez[#,1,2], Yang Zhao[#,2], Yoko Nishiga[3,4,5], Frank Peprah[6,7], Jiao Shen[7,8] , Gita C. Abhiraman[1,2], Masato Ogishi,[2] Chenyu Zhang[9], Justin Saco[10,11], Deepa Waghray[2], Mamatha Serasanambati[12], Leonel Torres[13], Brandon W. Simone[14], Leon Su[2], Steven C. Wilson[2], Aerin Yang[2], Qinli Sun[2], Lora Picton[2], Robert A. Saxton[2], Vidit Bhandarkar[15,16], Madeline J. Lee[1,17], Elizabeth Andrews[9], Hua Jiang[2], Matthias Obenaus[2], Michelle Yen[2], Tavus Atajanova[6,7], Catherine A. Blish[17,18], Stefani Spranger[15,16,20], E. John Wherry[13,21], Amanda Kirane[12], Antoni Ribas[11,22,23], David H. Raulet[9], Anusha Kalbasi[24,25,26], Stephanie K. Dougan[7,8], Michael Dougan[6,19], Julien Sage[4,5], K. Christopher Garcia[2,27,28*]

Corresponding author: kcgarcia@stanford.edu

The PDF file includes:

References #62-#70

Figs. S1 to S13

Reproducibility Checklist

References #62-#70:

62. J. Yodoi, K. Teshigawara, T. Nikaido, K. Fukui, T. Noma, T. Honjo, M. Takigawa, M. Sasaki, N. Minato, M. Tsudo, TCGF (IL 2)-receptor inducing factor(s). I. Regulation of IL 2 receptor on a natural killer-like cell line (YT cells). *J. Immunol.* **134**, 1623–1630 (1985).

63. O. Shalem, N. E. Sanjana, E. Hartenian, X. Shi, D. A. Scott, T. Mikkelson, D. Heckl, B. L. Ebert, D. E. Root, J. G. Doench, F. Zhang, Genome-scale CRISPR-Cas9 knockout screening in human cells. *Science* **343**, 84–87 (2014).

64. Y. Zhao, A. D. Bennett, Z. Zheng, Q. J. Wang, P. F. Robbins, L. Y. L. Yu, Y. Li, P. E. Molloy, S. M. Dunn, B. K. Jakobsen, S. A. Rosenberg, R. A. Morgan, High-affinity TCRs generated by phage display provide CD4+ T cells with the ability to recognize and kill tumor cell lines. *J. Immunol.* **179**, 5845–5854 (2007).

65. A. Nowak, R. Marlow, K. Ryan, J.-M. Lapointe, D. Sutton, A. Sharpe, L. Crook, J. A. Walker, E. Little, J. Peverill, A. Holberry-Brown, E. Wassell, R. McLaren-Jones, C. Cavanagh, A. V. Dobre, T. Baker, M. Clayton, N. A. Karp, M. Plugge, A. A. Thomas, S. J. Dovedi, S. I. Sitnikova, N. Burrows, A comprehensive welfare scoring system for graft versus host disease clinical assessment in humanised mouse models used for pharmaceutical research. *Front. Immunol.* **16**, 1617528 (2025).

66. C. J. Nicolai, N. Wolf, I.-C. Chang, G. Kirn, A. Marcus, C. O. Ndubaku, S. M. McWhirter, D. H. Raulet, NK cells mediate clearance of CD8+ T cell-resistant tumors in response to STING agonists. *Sci. Immunol.* **5**, eaaz2738 (2020).

67. M. Grange, G. Verdeil, F. Arnoux, A. Griffon, S. Spicuglia, J. Maurizio, M. Buferne, A.-M. Schmitt-Verhulst, N. Auphan-Anezin, Active STAT5 regulates T-bet and eomesodermin expression in CD8 T cells and imprints a T-bet-dependent Tc1 program with repressed IL-6/TGF-β1 signaling. *J. Immunol.* **191**, 3712–3724 (2013).

68. G. Verdeil, D. Puthier, C. Nguyen, A.-M. Schmitt-Verhulst, N. Auphan-Anezin, STAT5-mediated signals sustain a TCR-initiated gene expression program toward differentiation of CD8 T cell effectors. *J. Immunol.* **176**, 4834–4842 (2006).

69. A. V. Villarino, G. Sciumè, F. P. Davis, S. Iwata, B. Zitti, G. W. Robinson, L. Hennighausen, Y. Kanno, J. J. O'Shea, Subset- and tissue-defined STAT5 thresholds control homeostasis and function of innate lymphoid cells. *J. Exp. Med.* **214**, 2999–3014 (2017).

70. G. M. Wiedemann, S. Grassmann, C. M. Lau, M. Rapp, A. V. Villarino, C. Friedrich, G. Gasteiger, J. J. O'Shea, J. C. Sun, Divergent role for STAT5 in the adaptive responses of natural killer cells. *Cell Rep.* **33**, 108498 (2020).

Supplemental Figures

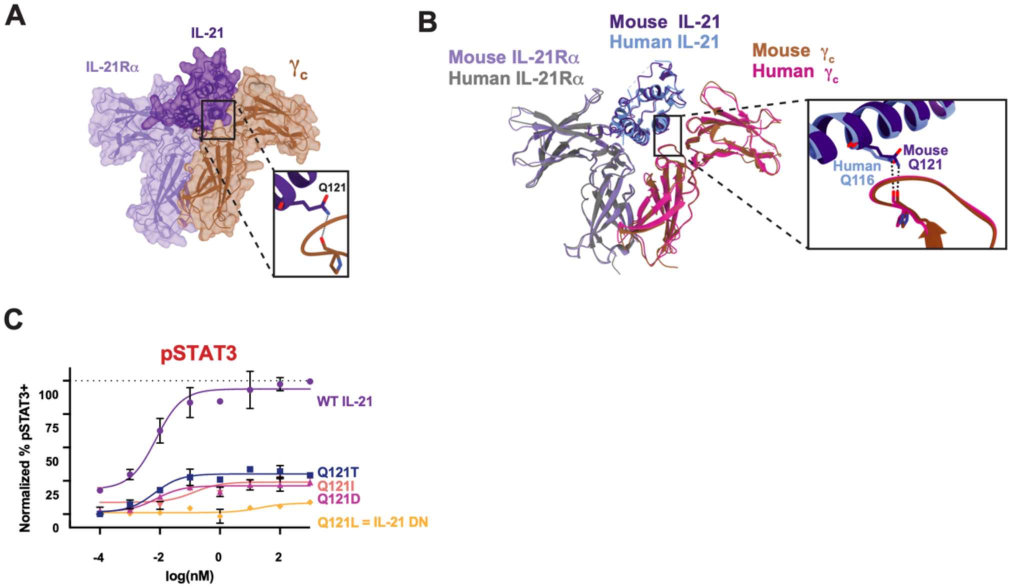

Fig. S1. Design of mouse IL-21 DN.

(**A**) Alphafold 3 model of mouse IL-21, IL-21Rα, and γ_c in complex with magnified view of IL-21 Q121 at the binding interface with γ_c. (**B**) Structural alignment of the human and predicted mouse IL-21 signaling complexes with magnification of human IL-21 Q116 and mouse IL-21 Q121. The alignment includes depictions of human IL-21 (blue), mouse IL-21 (dark purple), human IL-21Rα (gray), mouse IL-21Rα (light purple), human γ_c (pink), and mouse γ_c (brown). (**C**) pSTAT3 signaling of WT IL-21 versus IL-21 with mutations at Q121 on mouse T cells. Errors bars indicate mean ± standard deviation (SD) of duplicate or triplicate wells. MFI, mean fluorescence intensity. Results from one independent experiment.

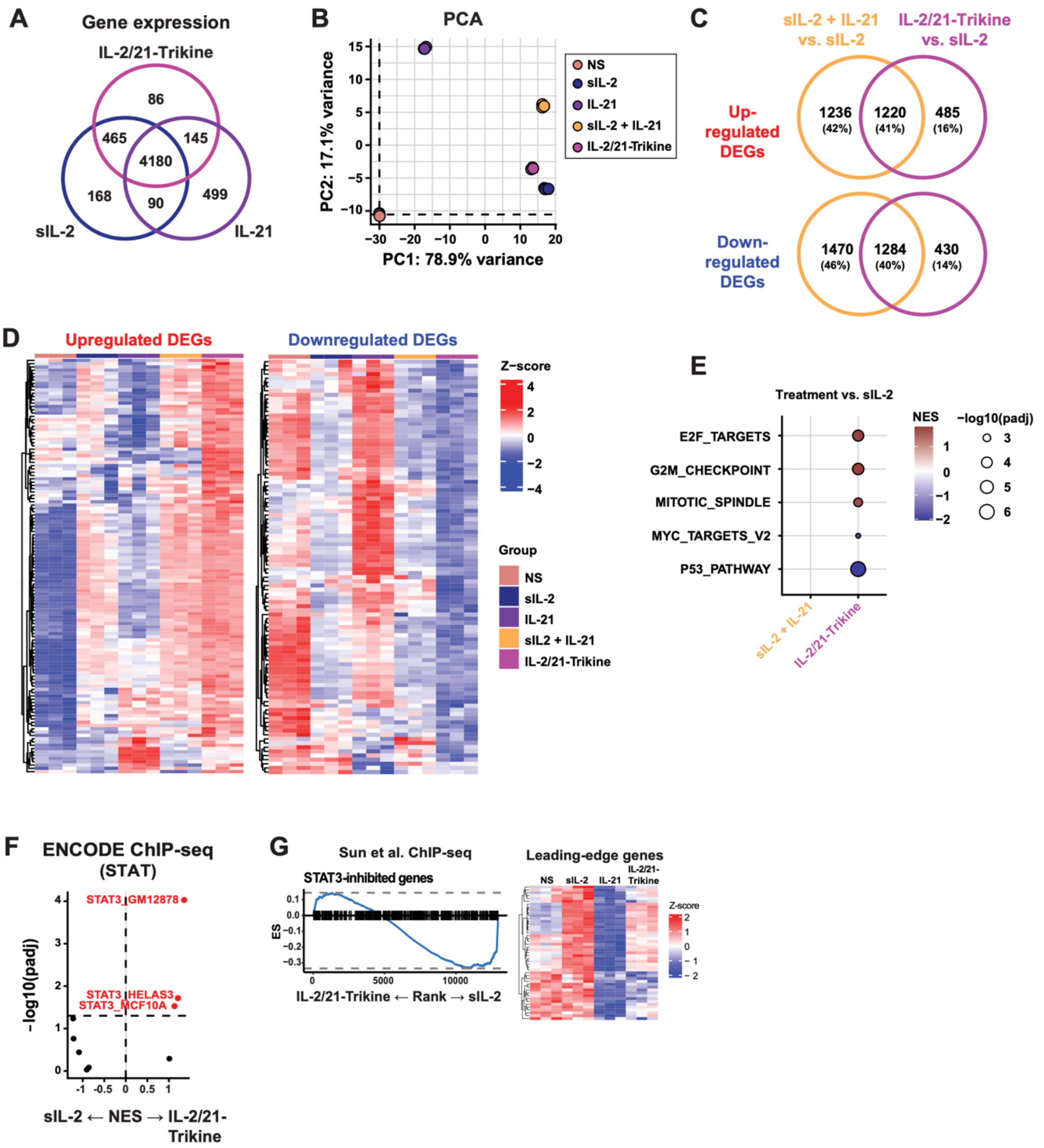

Fig. S2. Mouse-reactive IL-2/21-Trikine development and its differential gene expression program on mouse CD8+ T cells.

(**A**) Venn diagram displaying shared and unique expression of genes by mouse T cells cultured with 10 nM sIL-2, IL-21, or Trikine for 24 hours as determined by bulk RNA-seq. (**B**) Principal component analysis demonstrating variance in DEGs between mouse CD8+ T cells treated with no treatment, sIL-2, IL-21, sIL-2 + IL-21, or mouse-reactive IL-2/21-Trikine for 24 hours. (**C-D**) DEGs up- and down-regulated by sIL-2 + IL-21 and IL-2/21-Trikine in relation to sIL-2; (C) Venn

diagram representation of up- and downregulated DEGs, (D) heatmaps of Z-transformed normalized read counts of uniquely IL-2/21-Trikine up- (left) and downregulated (right) DEGs as identified in (C). (**E**) GSEA for DEGs between sIL-2 + IL-21 vs. sIL-2 and IL-2/21-Trikine vs. sIL-2 using Hallmark gene sets. (**F**) GSEA for DEGs between Trikine-treated vs IL2-treated animals using ENCODE ChIP-seq gene sets (n = 552 genesets, of which 9 are for STAT family TFs) obtained from the ChEA3 web portal (REF). Human gene symbols were converted into mouse gene symbols based on the NCBI homologene database (ftp://ftp.ncbi.nih.gov/pub/HomoloGene/build68/). Displayed are results for STAT family TFs. (**G**) Geneset enrichment analysis (GSEA) for DEGs between IL-2/21-Trikine-treated vs sIL2-treated mouse CD8+ T cells (left) and heatmap of leading-edge genes (right) as described in Fig. 2A. pSTAT3-inhibited genes, genes with stronger ChIP peaks in STAT3-KO mice. Results from one independent experiment containing 3 technical replicates.

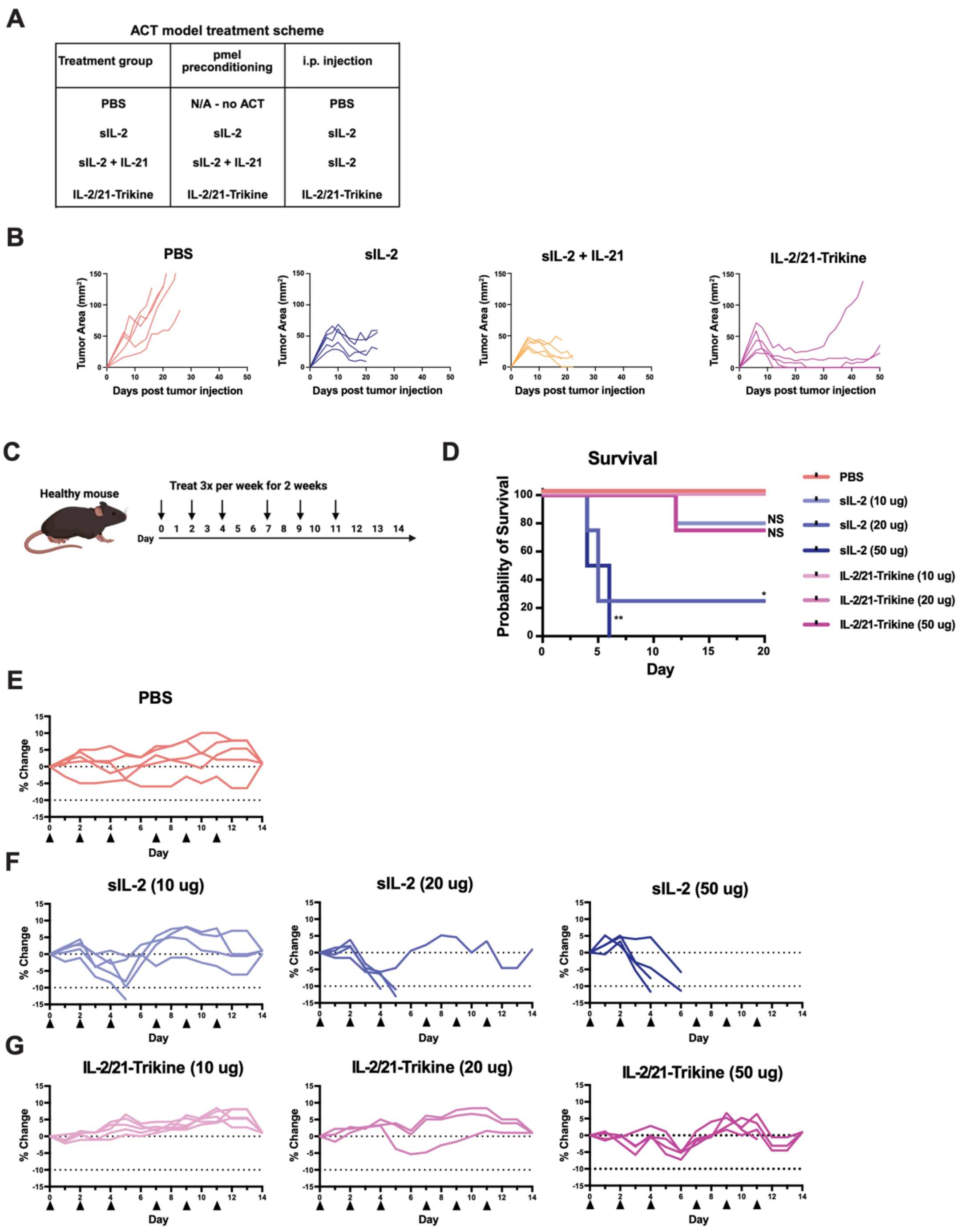

Fig. S3. IL-2/21-Trikine is well-tolerated in an ACT model and is 5x better tolerated than sIL-2 in healthy mice.

(**A**) Table displaying the ACT model treatment scheme. (**B**) Individual tumor growth curves divided by treatment received in experiment described in Fig. 2E-F. (**C-D**) Healthy C57BL/6 mice received 10 μg, 20 μg, or 30 μg functional doses of sIL-2 or IL-2/21-Trikine or PBS thrice weekly

for two weeks; the experimental timelines (C), survival curves (D). Survival data were analyzed using the log-rank (Mantel-Cox) and Gehan-Breslow Wilcoxon tests between individual treatment groups and the PBS group, n = 5 mice/group. (**E-G**) Individual weight change curves of mice receiving PBS (E) and varied dosages of sIL-2 (F) or IL-2/21-Trikine (G) over the course of the study described in (C). Arrows indicate treatment dates and curves end when mice met euthanasia criteria. All results from one independent experiment. Schematic in C created using BioRender.com.

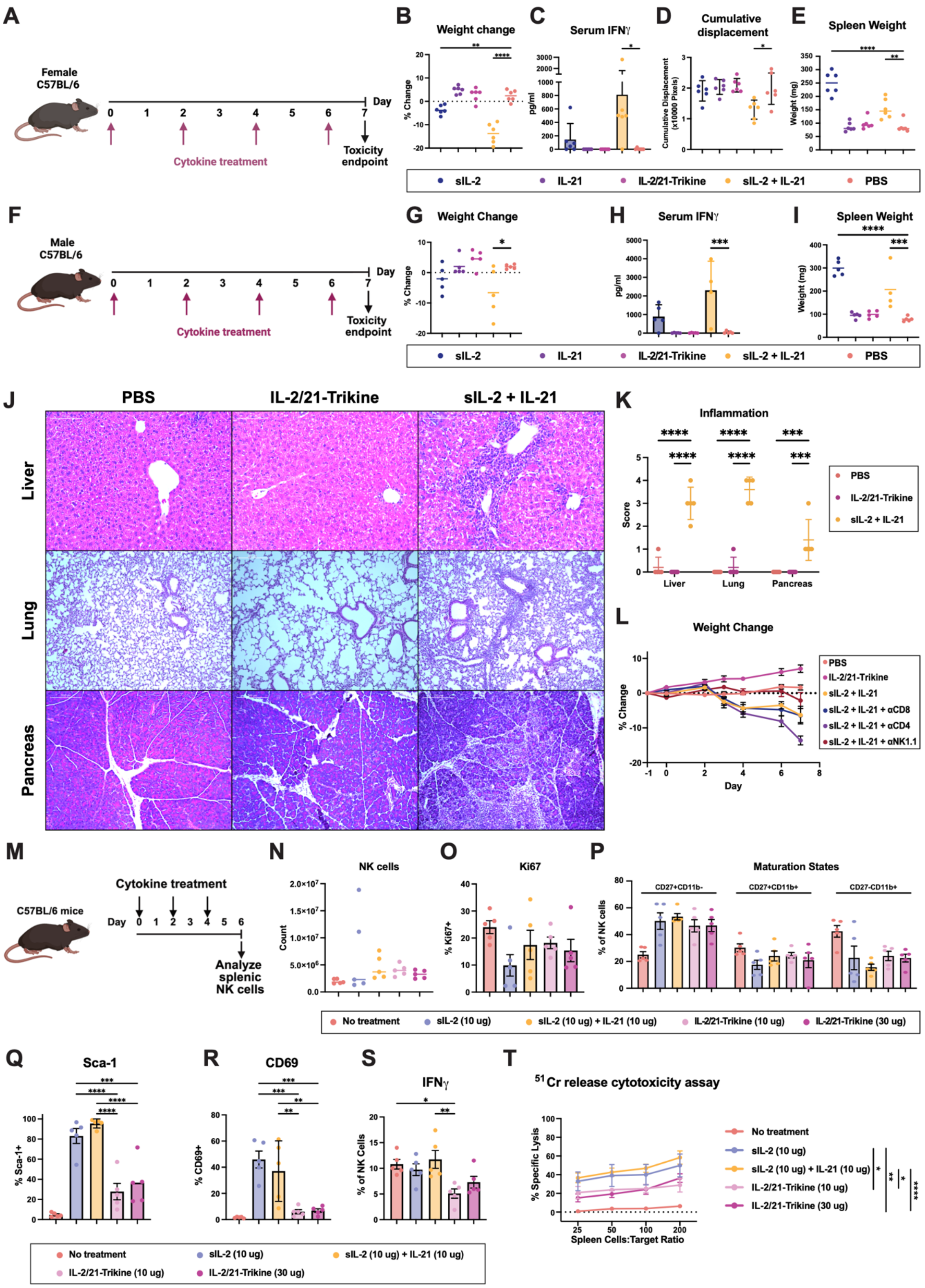
A
Female C57BL/6
Day
0 1 2 3 4 5 6 7
Cytokine treatment
Toxicity endpoint
B
Weight change
% Change
C
Serum IFNγ
pg/ml
D
Cumulative displacement
Cumulative Displacement (x10000 Pixels)
E
Spleen Weight
Weight (mg)
sIL-2
IL-21
IL-2/21-Trikine
sIL-2 + IL-21
PBS
F
Male C57BL/6
G
Weight Change
H
Serum IFNγ
I
Spleen Weight
J
PBS
IL-2/21-Trikine
sIL-2 + IL-21
Liver
Lung
Pancreas
K
Inflammation
Score
L
Weight Change
sIL-2 + IL-21 + αCD8
sIL-2 + IL-21 + αCD4
sIL-2 + IL-21 + αNK1.1
Day
M
C57BL/6 mice
Cytokine treatment
Analyze splenic NK cells
N
NK cells
Count
O
Ki67
% Ki67+
P
Maturation States
CD27+CD11b-
CD27+CD11b+
CD27-CD11b+
% of NK cells
No treatment
sIL-2 (10 ug)
sIL-2 (10 ug) + IL-21 (10 ug)
IL-2/21-Trikine (10 ug)
IL-2/21-Trikine (30 ug)
Q
Sca-1
% Sca-1+
R
CD69
% CD69+
S
IFNγ
% of NK Cells
T
51Cr release cytotoxicity assay
% Specific Lysis
Spleen Cells:Target Ratio

Fig. S4. IL-2/21-Trikine avoids toxicity seen by sIL-2 + IL-21 treatment via differential effects on NK cells.

(**A-E**) Healthy female C57BL/6 mice were dosed with 10 µg functional dose of sIL-2, IL-21, sIL-2 + IL-21, IL-2/21-Trikine or PBS every other day for four total doses (n = 5-6 mice); experimental scheme Results from representative of 2 independent experiments. (A), percent weight change at study endpoint (B), serum IFN-γ measurements (C), cumulative displacement of mice when tracked for one minute (D), spleen weight at study endpoint (E). (**F-I**) Healthy male C57BL/6 mice were treated as described in (A); experimental scheme (F), percent weight change at study endpoint (G), serum IFN-γ measurements (H), spleen weight at study endpoint (I). Results from one independent experiment. (**J-K**) Histology results among mice in the PBS, IL-2/21-Trikine, and sIL-2 + IL-21 treatment groups in (A); representative images (J), blinded inflammation scoring (K).The histology sections were imaged at a magnification of 20x (liver) or 10x (lung and pancreas) and scale bars in the top right of each image represent 100 µm (liver) or 200 µm (lung and pancreas). Results from one independent experiment. (**L**) Percent weight change over course of study as described in Fig. 2G. Errors bars indicate mean ± standard deviation (SD) of n = 10 mice/group. Results are combined from 2 independent experiments. (**M-T**) Healthy C57BL/6 mice were dosed with 10 µg or 30 µg functional dose of sIL-2, IL-21, sIL-2 + IL-21, IL-2/21-Trikine or PBS every other day for four total doses (n = 5 mice); experimental timeline (M) , NK cell count (N), percentage of Ki67+ NK cells (O), percentage of NK cells at indicated maturation states (P), percentage of Sca-1+ NK cells (Q), percentage of CD69+ NK cells (R), percentage of IFN-γ+ NK cells (S), percentage of specific lysis of target cells by isolated NK cells in a ^{51}Cr release assay (T). Errors bars indicate mean ± standard deviation (SD) of n = 5 mice/group. Results from one independent experiment. Schematics in A, F, and M created using BioRender.com.

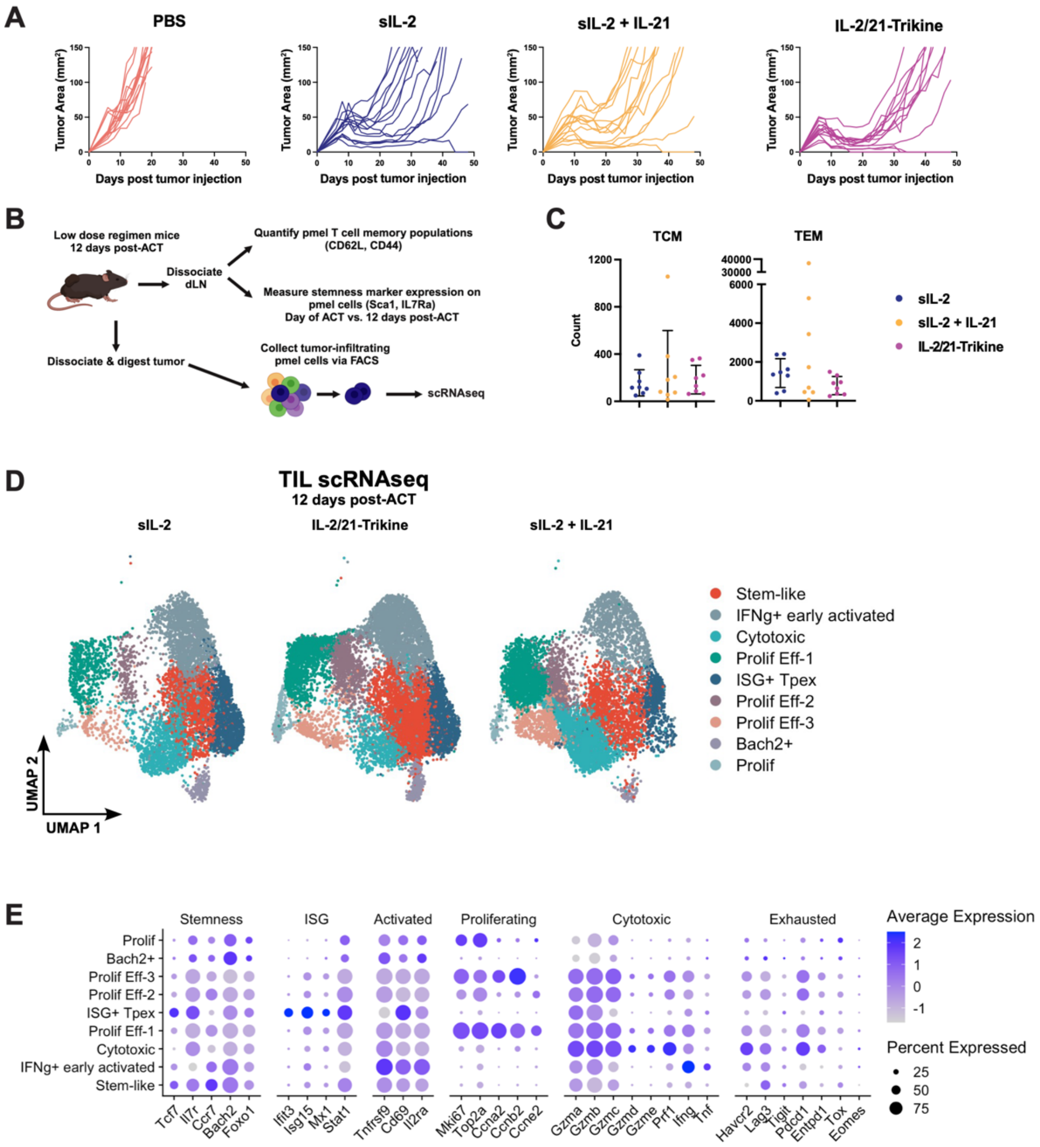

Fig. S5. IL-2/21-Trikine enhances anti-tumor efficacy and T cell stemness in ACT in the B16F10 tumor model.

(**A**) Individual tumor growth curves divided by treatment received in experiment described in Fig. 2I-J. Results are combined from 2 independent experiments. (**B**) Ex vivo TIL analysis scheme resulting in data presented in Fig. 2M-P and S6C-E. (**C**) Raw counts of TCM and TEM cells isolated from dLN as in (B) and represented as percentages of Thy1.1+CD8+ cells in Fig. 2M. Results representative of 2 independent experiments. (**D**) UMAP by treatment group as in Fig. 2P. (**E**) Key differentially expressed genes defining transcriptional clusters as in Fig. 2P.

scRNAseq data is from one independent experiment. Schematic in B created using BioRender.com.

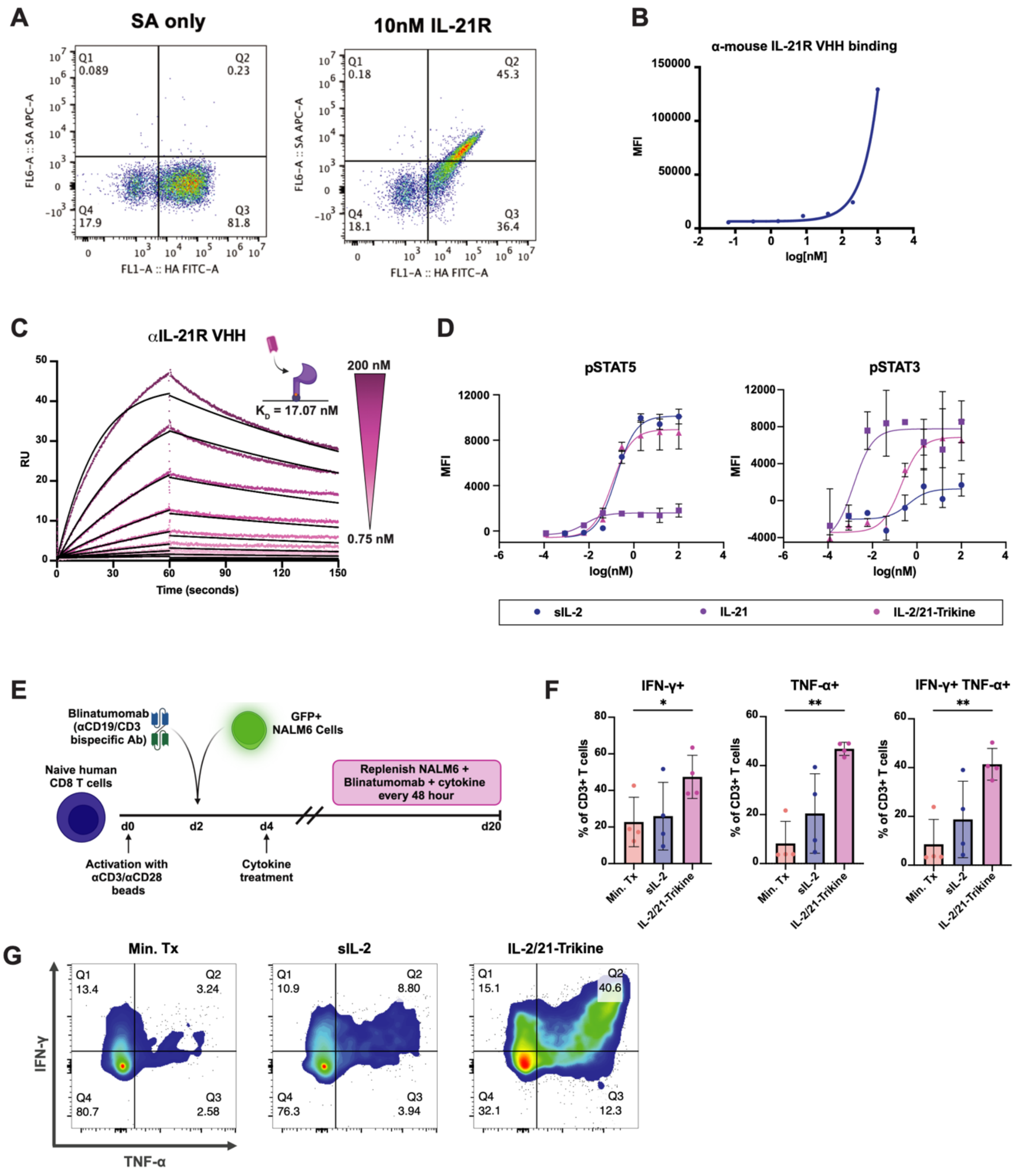

Fig. S6. Human-reactive IL-2/21-Trikine development and characterization in a repeated stimulation assay.

(**A**) Binding of streptavidin (left) and 10 nM biotinylated IL-21Rα (right) to yeast expressing selected anti-IL-21Rα VHH. (**B**) Dose-dependent binding of selected anti-IL-21Rα VHH to YT-1 cells. (**C**) Surface plasmon resonance (SPR) sensorgram determines binding affinity of anti-IL-21Rα VHH for human IL-21Rα. RU = Response Units. (**D**) Dose-dependent pSTAT signaling of

sIL-2, IL-21, and human-reactive IL-2/21-Trikine on (human NK cell-derived) YT-1 cells. MFI, mean fluorescence intensity. (**E**) Schematic of chronic *in vitro* stimulation experiment using T cells isolated from 4 human donors. (**F**) Percentage of CD3+ T cells positive for IFN-γ (left), positive for TNF (middle), or double positive for IFN-γ and TNF (right) on day 25 of experiment described in (S1E) Error bars represent mean ± SD and data were analyzed by one-way ANOVA with Tukey's multiple comparison relative to Min. Tx, n = 4 human donors. (**G**) Representative plots of data in (S1F). Schematics in C and E created using BioRender.com. All results are representative of one (A-C) or 2 (D-G) independent experiments.

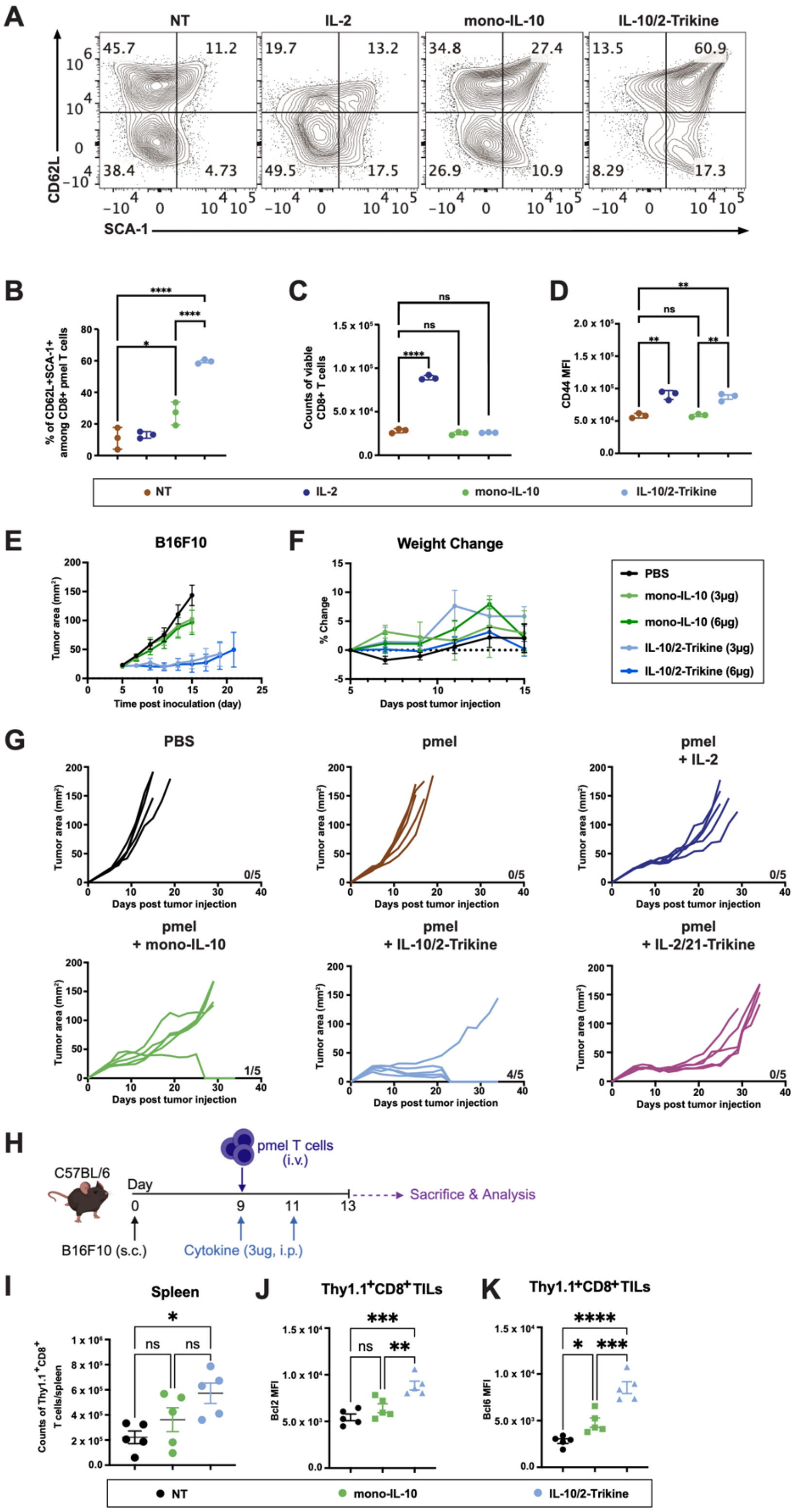
A
NT
IL-2
mono-IL-10
IL-10/2-Trikine
45.7
11.2
38.4
4.73
19.7
13.2
49.5
17.5
34.8
27.4
26.9
10.9
13.5
60.9
8.29
17.3
CD62L
SCA-1
B
% of CD62L+SCA-1+ among CD8+ pmel T cells
C
Counts of viable CD8+ T cells
D
CD44 MFI
NT
IL-2
mono-IL-10
IL-10/2-Trikine
E
B16F10
Tumor area (mm²)
Time post inoculation (day)
F
Weight Change
% Change
Days post tumor injection
PBS
mono-IL-10 (3μg)
mono-IL-10 (6μg)
IL-10/2-Trikine (3μg)
IL-10/2-Trikine (6μg)
G
PBS
pmel
pmel + IL-2
pmel + mono-IL-10
pmel + IL-10/2-Trikine
pmel + IL-2/21-Trikine
Tumor area (mm²)
Days post tumor injection
0/5
0/5
0/5
1/5
4/5
0/5
H
C57BL/6
Day
pmel T cells (i.v.)
Sacrifice & Analysis
B16F10 (s.c.)
Cytokine (3ug, i.p.)
I
Spleen
Counts of Thy1.1+CD8+ T cells/spleen
J
Thy1.1+CD8+ TILs
Bcl2 MFI
K
Thy1.1+CD8+ TILs
Bcl6 MFI
NT
mono-IL-10
IL-10/2-Trikine

Fig. S7. IL-10/2-Trikine preserves T cell stemness and enhances antitumor efficacy of ACT in the B16F10 tumor model.

(**A-D**), Activated pmel CD8$^+$ T cells were cultured with the indicated cytokines (10 nM) for 48 hours, followed by flow cytometry analysis (n = 3). (A-B), Shown are representative flow cytometry plots (A) and frequencies (B) showing SCA-1+CD62L+ cells among pmel CD8$^+$ T cells. (C), Counts of viable CD8$^+$ T cells. (D), Mean fluorescence intensity (MFI) of CD44**.** Results representative of 3 independent experiments. (**E-F**) C57BL/6 mice bearing established subcutaneous B16F10 tumors received intraperitoneal injections of PBS, mono-IL-10, or IL-10/2-Trikine (equivalent to 3 or 6 μg functional IL-10) starting on day 5 and administered every other day until day 19 (n = 5 per group). Shown are the average tumor growth curves (E) and relative body weight (F). Results from one independent experiment. (**G**), Experimental setting is described in Fig. 4D. Shown are individual tumor growth curves. Indicated are numbers of tumor-free mice among the total number of mice in the group. Results representative of 2 independent experiments. (**H-K**), C57BL/6 mice bearing established s.c. B16F10 tumors received i.v. ACT of pmel CD8$^+$ T cells (5×10^6) on day 9, followed by i.p. injections of cytokine (3 μg functional cytokine) or left NT on days 9 and 11 (n = 5 animals). Mice were sacrificed on day 13, tumors and spleens were collected for flow cytometry analysis. (H), The experimental timeline. (I), Counts of Thy1.1+CD8+ TILs in spleens. (J), MFI of Bcl-2 in Thy1.1+CD8+ TILs. (K), MFI of Bcl-6 in Thy1.1+CD8+ TILs. Results representative of 2 independent experiments. All data represent mean ± s.e.m. and are analyzed by one-way ANOVA with Tukey's post-test (B to D, I to K). Schematic in F created using BioRender.com.

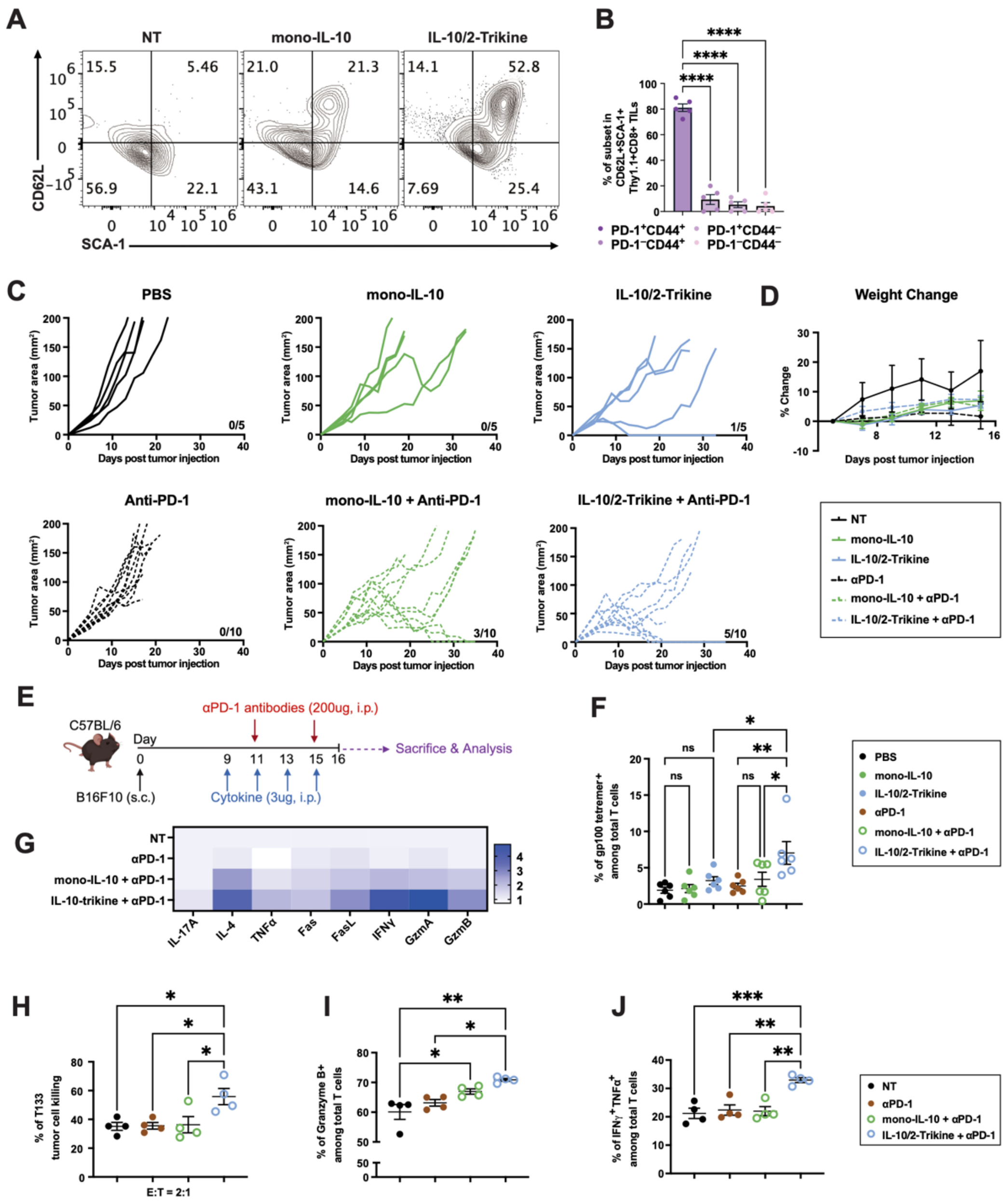

Fig. S8. IL-10/2-Trikine potentiates anti–PD-1 therapy in murine B16F10 melanoma and human tumor organoid models.

(**A-B**), Experimental setting is described in Fig. S7H. C57BL/6 mice bearing established s.c. B16F10 tumors received i.v. ACT of pmel CD8+ T cells (5×10^6) on day 9, followed by i.p. injections of cytokine (3 µg functional cytokine) or left NT on days 9 and 11 (n = 5 animals). Mice were sacrificed on day 13, tumors were collected for flow cytometry analysis. (A), Representative flow cytometry plots showing SCA-1+CD62L+ cells among Thy1.1+CD8+ TILs.

(B), Frequencies of subsets among SCA-1+CD62L+ Thy1.1+CD8+ TILs. Results representative of 2 independent experiments. (**C-D**), Experimental setting is described in Fig. 4M. (C), Shown are individual tumor growth curves. Indicated are numbers of tumor-free mice among the total number of mice in the group. (D), Relative body weight. Results representative of 2 independent experiments. (**E-F**), C57BL/6 mice bearing B16F10 tumors were administered i.p. mono-IL-10, IL-10/2-Trikine (3 µg functional cytokine per dose), or PBS every other day from day 9 to day 15. Mice also received i.p. injections of anti-PD-1 antibody (200 µg per injection) on days 11, and 15. On day 16, mice were sacrificed, and tumors were collected for flow cytometry analysis (n = 6 animals). (E), The experimental timeline. (F), Frequencies of gp100 tetramer+ cells among total T cells in tumors. Results representative of 2 independent experiments. (**G**), Relative cytokine secretion levels in tumor organoid supernatants following the indicated treatments. Results representative of 2 independent experiments. (**H-J**), Autologous peripheral blood T cells were co-cultured with patient-derived tumor cells at an effector-to-target (E:T) ratio of 2:1 for 48 hours. Cells were then harvested for flow cytometry analysis. (H), Percentage of T133 tumor cell killing. (I), Frequency of Granzyme B^+ cells among total T cells. (J), Frequency of IFN-γ+TNF-α+ cells among total T cells. Results representative of 2 independent experiments. All data represent mean ± s.e.m. and are analyzed by one-way ANOVA with Tukey's post-test (B, F, H to J). Schematic in E created using BioRender.com.

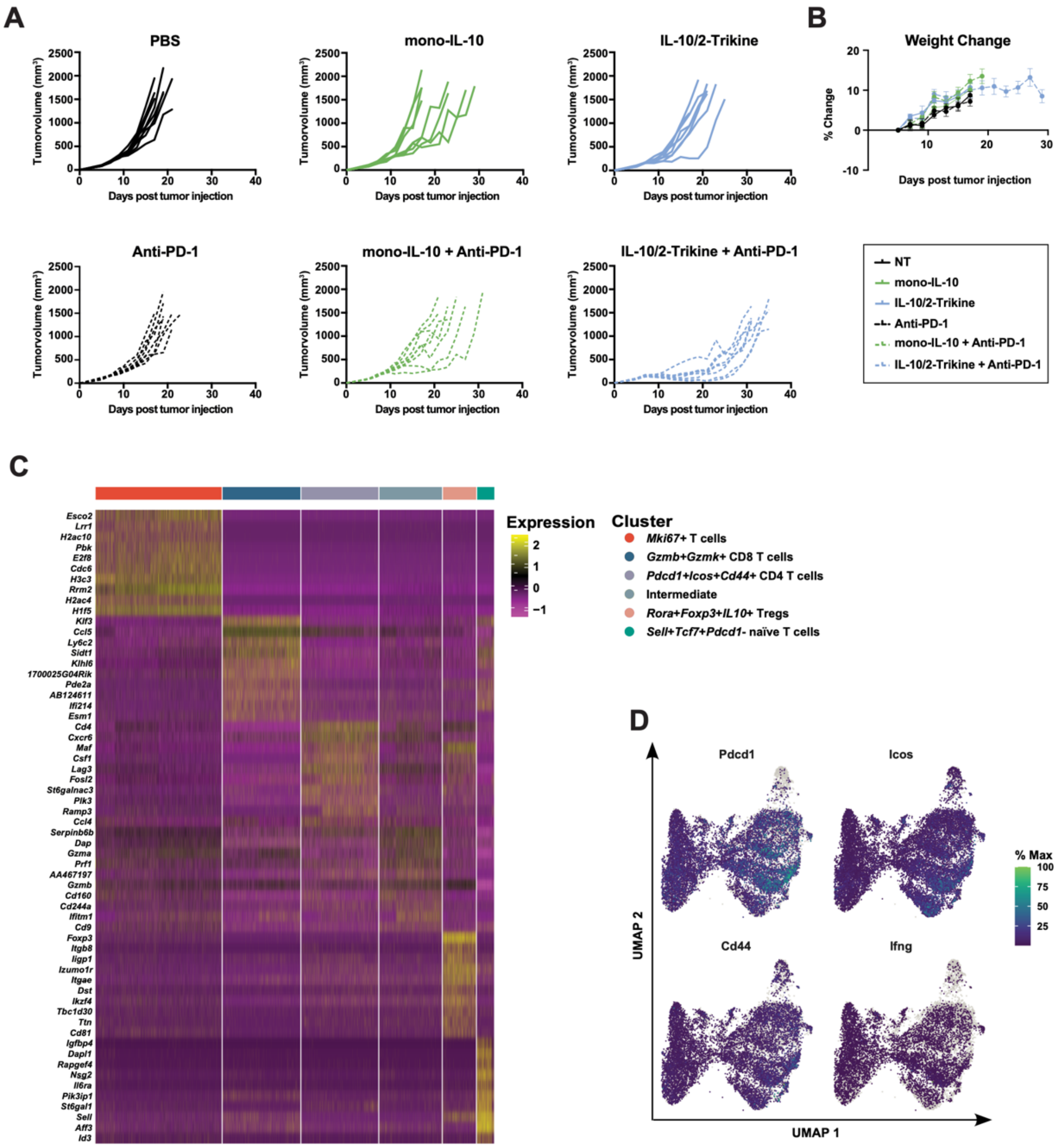

Fig. S9. IL-10/2-Trikine enhances the efficacy of anti–PD-1 therapy in the KP1 SCLC model. (**A-D**), Experimental setting is described in Fig. 5A and (A), Shown are individual tumor growth curves. (B), Relative body weight. (D), Heatmap of differentially expressed genes defining the identified T cell clusters. (E), UMAP showing the expression levels of *Pdcd1*, *Dd44*, *Icos*, and *Ifng*. Results representative of one (C-D) or 2 (A-B) independent experiments. All data in (A-B) represent mean ± s.e.m.

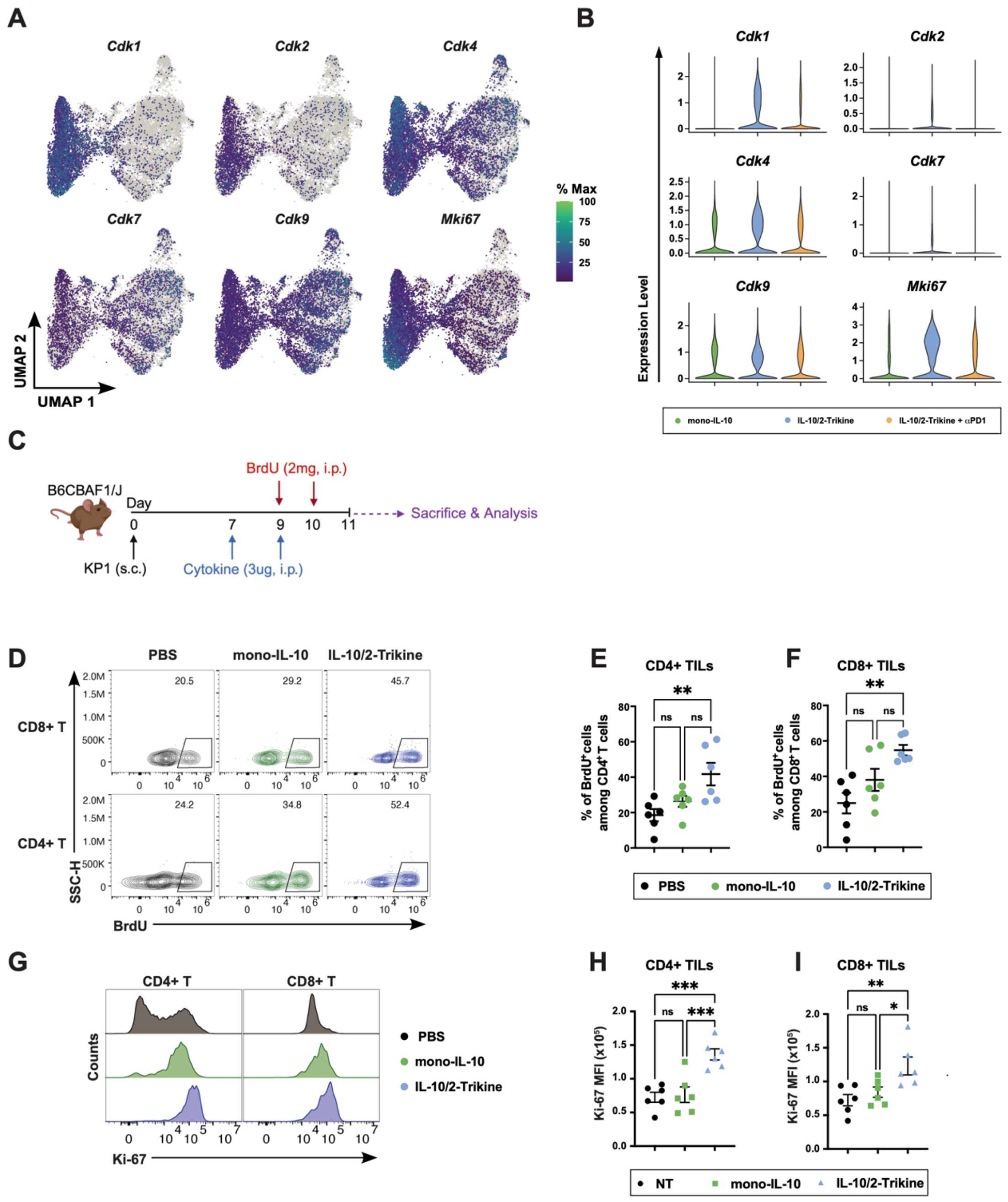

Fig. S10. IL-10/2-Trikine enriches proliferating T cells in KP1 tumors.

(**A-B**), The experimental setting is described in Fig. 5A. Shown are the UMAP (A) and violin plots (B) depicting the expression levels of cell cycle-associated genes. Results from one independent experiment. (**C-I**), B6129SF1/J mice bearing KP1 tumors received i.p. injections of mono-IL-10, IL-10/2-Trikine (3 µg functional cytokine per dose), or PBS on days 7 and 9. Bromodeoxyuridine (BrdU; 2 mg per injection) was administered i.p. on days 9 and 10. On day 11, mice were

sacrificed, and tumors were collected for flow cytometry analysis. (C), The experimental timeline. (D), Representative flow cytometry plots showing BrdU+ cells among CD4+ (top) and CD8+ (bottom) T cells. (E and F), Average frequencies of BrdU+ cells among CD4+ (E) and CD8+ (F) T cells. **(G),** Representative flow cytometry histograms showing Ki-67 expression level. (H-I), Ki-67 MFI in CD4+ T cells (H) and CD8^{+} T cells (I). Results representative of 2 independent experiments. All data represent mean ± s.e.m. and are analyzed by one-way ANOVA with Tukey's post-test (E, F, H-I). Schematic in C created using BioRender.com.

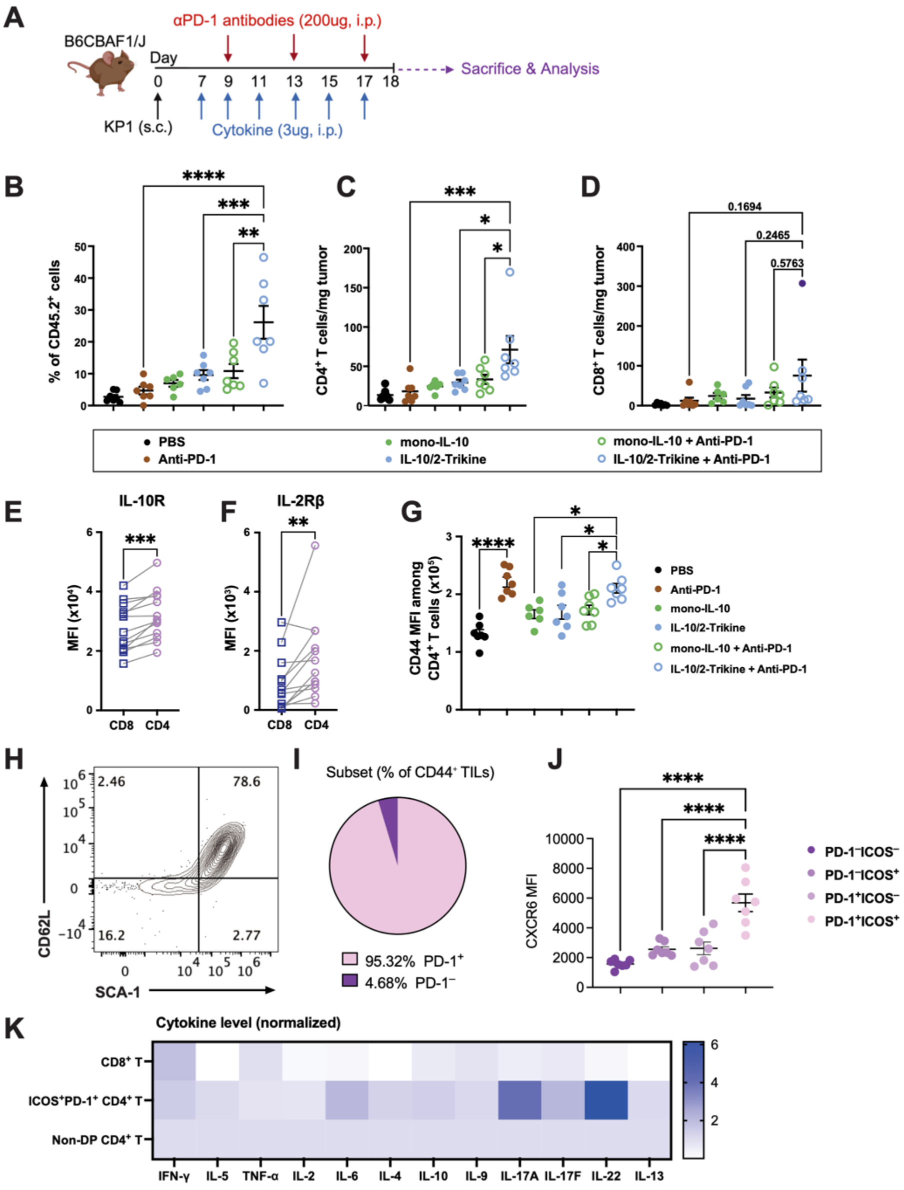

Fig. S11. IL-10/2-Trikine enriches PD-1⁺ICOS⁺ CD4⁺ Th cells in the KP1 tumor model. (**A-K**), B6129SF1/J mice bearing KP1 tumors were administered i.p. mono-IL-10, IL-10/2-Trikine (3 µg functional cytokine per dose), or PBS every other day from day 7 to day 17. Mice also received i.p. injections of anti-PD-1 antibody (200 µg per dose) on days 9, 13, and 17. On day 18, mice were sacrificed, and tumors were collected for flow cytometry analysis (n = 7 animals). (A), The experimental timeline. (B), Frequencies of CD45.2+ cells among single live cells in tumors. (C), Counts of CD4+ T cells per mg of tumor tissue. (D), (E), IL-10R MFI in CD4+ and CD8+ TILs. (F), IL-2Rβ MFI in CD4+ and CD8+ TILs. (G), CD44 MFI in CD4+ TILs of each treatment

group. (H), Representative flow cytometry plots showing co-expression of PD-1 and CD44 in TILs. (I), Average frequencies of CD44+ cells among PD-1+ and PD-1- T cells. (J), CXCR6 MFI in PD-1/ICOS quadrant-gated subsets. (K), Normalized cytokine secretion levels across T cell subsets. Results representative of 2 independent experiments. All data represent mean ± s.e.m. and are analyzed by two-tailed Student's t-test (E, F) or one-way ANOVA with Tukey's post-test (B to D, G, J). Schematic in A created using BioRender.com.

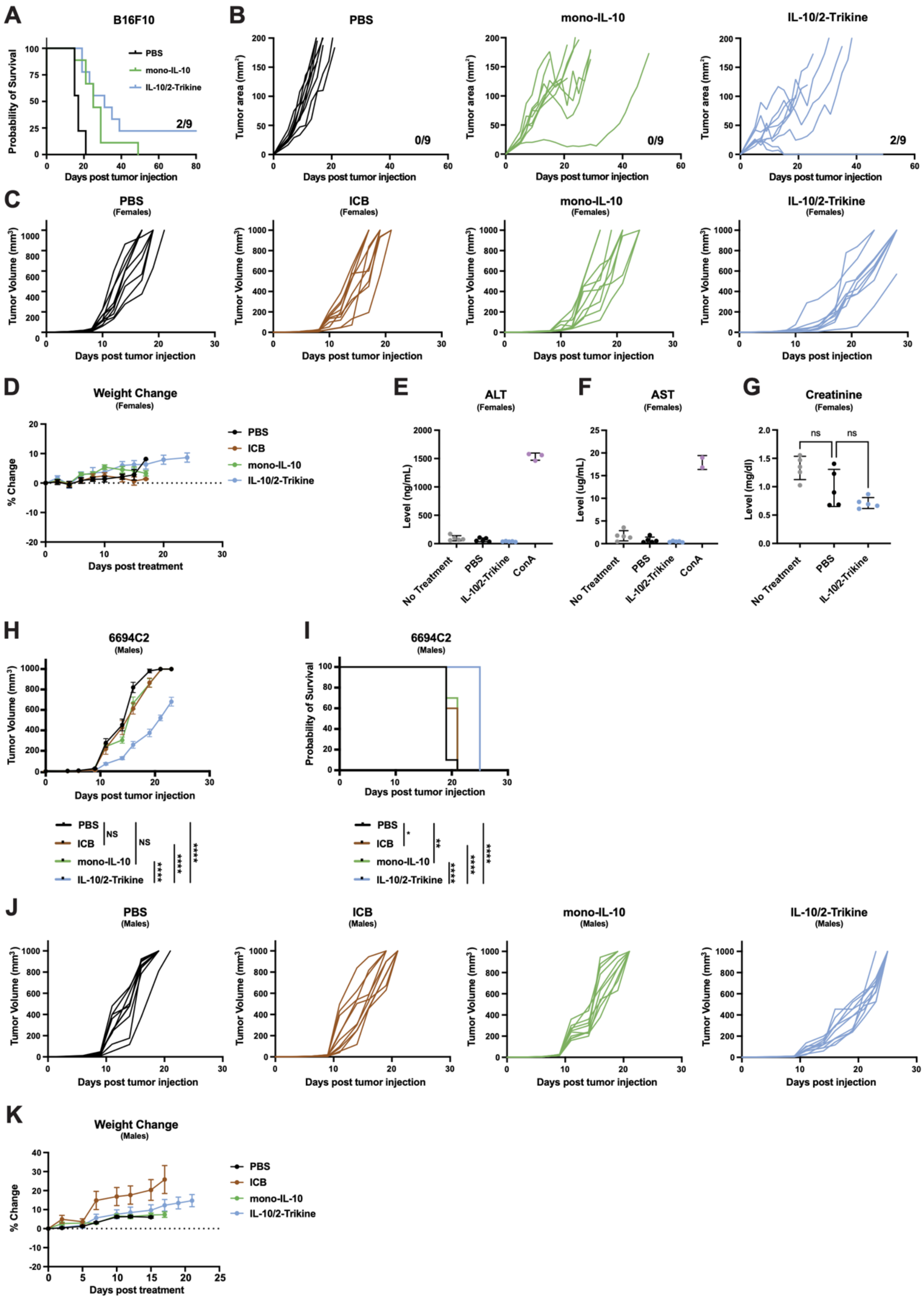
A
B16F10
PBS
mono-IL-10
IL-10/2-Trikine
2/9
Probability of Survival
Days post tumor injection
B
PBS
mono-IL-10
IL-10/2-Trikine
0/9
0/9
2/9
Tumor area (mm²)
Days post tumor injection
C
PBS (Females)
ICB (Females)
mono-IL-10 (Females)
IL-10/2-Trikine (Females)
Tumor Volume (mm³)
Days post tumor injection
Days post injection
D
Weight Change (Females)
% Change
Days post treatment
PBS
ICB
mono-IL-10
IL-10/2-Trikine
E
ALT (Females)
Level (ng/mL)
F
AST (Females)
Level (ug/mL)
No Treatment
PBS
IL-10/2-Trikine
ConA
G
Creatinine (Females)
Level (mg/dl)
ns
ns
H
6694C2 (Males)
Tumor Volume (mm³)
Days post tumor injection
PBS
ICB
mono-IL-10
IL-10/2-Trikine
NS
NS
****
****
****
I
6694C2 (Males)
Probability of Survival
Days post tumor injection
*
**
****
****
****
J
PBS (Males)
ICB (Males)
mono-IL-10 (Males)
IL-10/2-Trikine (Males)
Tumor Volume (mm³)
Days post tumor injection
Days post injection
K
Weight Change (Males)
% Change
Days post treatment

Fig. S12. IL-10-trikine monotherapy inhibits the growth of murine melanoma and pancreatic cancer models.

(**A-B**), Experimental setting is described in Fig. 6A. (B), Shown are individual tumor growth curves. Indicated are numbers of tumor-free mice among the total number of mice in the group. (B), Survival curves. (**C-G**) Experimental setting is described in Fig. 6M and performed on female mice; individual tumor growth curves (C), relative weight change (D), ALT (E), AST (F), and Creatinine (G) levels in the blood 5 days post treatment. (**H-K**) Experimental setting is described in Fig. 6M and performed on male mice; cumulative tumor growth curves (H), survival curves (I), individual tumor growth curves (J), relative weight change (K). All data represent mean ± s.e.m (D, H, K) or ± SD (E-G) and are analyzed by one way (E-G) or two-way (H) ANOVA with Tukey's post-test. Results representative of one (H-K) or 2 (A-I) independent experiments.

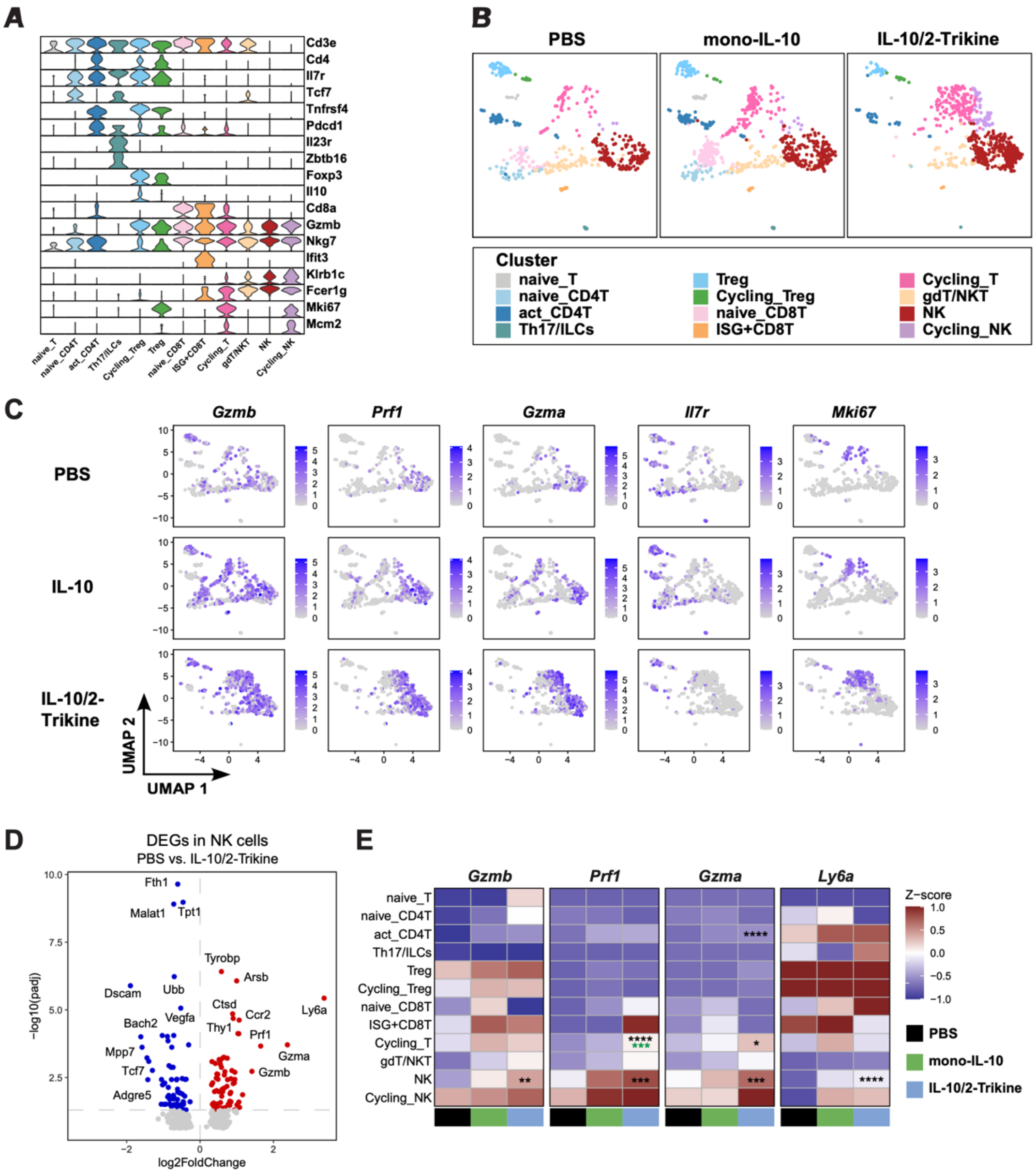

Fig. S13. IL-10/2-Trikine increases expression of cytotoxic markers in NK cells

(**A-E**) T and NK cell subcluster from Figure 6P. Violin plots showing log-normalized expression of the indicated marker genes across clusters (A). UMAPs split by treatment groups (B). UMAPs colored by expression scores of selected genes (*Gzmb*, *Prf1*, *Gzma*, *Il7r*, and *Mki67*) and split by treatment (C). Volcano plot of differentially expressed genes (DEGs) in all tumor-infiltrating CD3+ T and NK cells comparing IL-10/2-Trikine with PBS (D). Heatmap showing z-scaled expression of selected STAT5-driven genes across treatments and cell clusters (E). Statistical significance was assessed by Wilcoxon test. Black asterisks indicate comparisons between PBS

and IL-10/2-Trikine; green asterisks indicate comparisons between IL-10 and IL-10/2-Trikine. *P < 0.05, **P < 0.01, ***P < 0.001, ****P < 0.0001. Data from one independent experiment.
